# Genome-wide identification of *EuUSPs* in *Eucommia ulmoides* and the role of *EuUSP16* in rubber biosynthesis

**DOI:** 10.3389/fpls.2025.1655155

**Published:** 2025-08-20

**Authors:** Shangmei Long, Jiang Yang, Hongling Wang, Xi Chen, De-gang Zhao, Yichen Zhao

**Affiliations:** ^1^ College of Life Sciences, College of Tea Sciences, The Key Laboratory of Plant Resources Conservation and Germplasm Innovation in Mountainous Region (Ministry of Education), Guizhou University, Guiyang, China; ^2^ Plant Conservation and Breeding Technology Center, Institute of Crop Germplasm Resources, Guizhou Academy of Agricultural Sciences, Guiyang, China

**Keywords:** *Eucommia ulmoides* Oliv., EuUSPs gene family, environmental stress, rubber biosynthesis, EuDof

## Abstract

*Eucommia ulmoides* Oliv., a Tertiary period relict tree species endemic to China, is a rubber-producing plant valued for both medicinal and edible applications. *E.ulmoides* rubber is a high-quality natural rubber prized for its excellent elasticity, abrasion resistance, and insulation properties, leading to broad industrial applications. Previous research identified the *EuUSP16* gene, encoding a protein containing *E.ulmoides* rubber particle protein peptides. While overexpression of *EuUSP16* in tobacco enhanced drought tolerance, its role in *E.ulmoides* rubber biosynthesis remained undefined. In this study, we identified 29 *EuUSP* genes at the whole-genome level in *E.ulmoides*. Following low-temperature and drought treatments, the expression level of the *EuUSP16* gene was found to be positively correlated with changes in rubber content (*p<0.05*), suggesting its potential regulatory role in rubber synthesis. In *E.ulmoides* subjected to Agrobacterium-mediated *EuUSP16* gene overexpression or silencing, the expression levels of key *E.ulmoides* rubber biosynthesis enzyme genes, such as *EuFPS1*, exhibited corresponding increases and decreases, respectively. Furthermore, rubber content in *EuUSP16*-overexpressing callus increased by 254.51% compared to wild-type callus. These findings indicate that *EuUSP16* regulates *E.ulmoides* rubber biosynthesis by modulating the expression of these genes. The 1,967 bp promoter region upstream of the *EuUSP16* ATG start codon contains several responsive elements, including MBS (MYB-binding site; CAACTG), LTR (low-temperature responsive element; CCGAAA), ABRE (ABA-responsive element; ACGTG), and a Dof transcription factor binding motif (AAAG). Promoter activity assays showed that *EuUSP16* promoter activity was induced by low temperature and drought but repressed by abscisic acid (ABA) treatment. Furthermore, using yeast one-hybrid screening, we identified a Cys2-Cys2 zinc finger domain-containing transcription factor, designated EuDof. Interaction analysis revealed that the EuDof transcription factor enhances the activity of the *EuUSP16* promoter. The binding of EuDof to the *EuUSP16* promoter was enhanced under low temperature and drought stress but inhibited by ABA. Collectively, this study provides crucial insights into the regulatory mechanism of *EuUSP16* in *E.ulmoides* rubber biosynthesis.

## 1 Introduction

*Eucommia ulmoides* Oliv. is a unique Tertiary relict tree species endemic to China, valued for over a millennium for its medicinal properties and, critically, as the source of *E.ulmoides* rubber (Wei et al., 2021; Zhu and Sun, 2018). This natural rubber, chemically defined as trans-1,4-polyisoprene (TPI), serves as the structural isomer of the *cis*-1,4-polyisoprene (CPI) from *Hevea brasiliensis* (Wei et al., 2021). Derived from secondary metabolism, *E.ulmoides* rubber is a vital natural polymer material with significant applications in aerospace, national defense, healthcare, and other advanced industries (Du et al., 2011). Its biosynthesis originates from isopentenyl pyrophosphate (IPP), produced via the mevalonic acid (MVA) or methylerythritol phosphate (MEP) pathways, central to isoprenoid metabolism which also generates terpenoids and other crucial bioactives (Miziorko, 2011; Men et al., 2019; Rohmer, 1999).

Universal Stress Proteins (USPs) represent a class of highly conserved, stress-induced proteins ubiquitous across diverse organisms (Ren et al., 2022). They play significant roles in plant responses to abiotic stresses. Characterized by a conserved C-terminal UspA domain (140–160 amino acids), USP proteins interact with various functional motifs, enabling diverse functions including substance transport, signal transduction, cell defense, and antioxidant responses (Aravind et al., 2002; Gustavsson et al., 2002). Numerous studies demonstrate their importance in stress tolerance, for instance, specific USP genes modulate salt tolerance in *Arabidopsis thaliana*, enhance growth and ion homeostasis under stress in transgenic tobacco, and overexpression of the *VyUSPA3* gene can improve drought tolerance in Chinese wild grapevine (*Vitis yeshanensis*), often interacting with hormone signaling (e.g., ethylene, abscisic acid), reactive oxygen species (ROS) scavenging, and ubiquitination pathways (Bhuria et al., 2022; Cui et al., 2022; Udawat et al., 2016).

While the isoprenoid metabolic pathway underpinning *E.ulmoides* rubber synthesis is known to be modulated by environmental stresses, the specific molecular regulators linking stress perception to enhanced rubber accumulation remain poorly understood. Intriguingly, our laboratory previously identified that the amino acid sequence of EuUSP16 protein contains a conserved rubber particle protein motif (-WALDNLADKGDTLYVLHLK-) (Hu and Zhao, 2024). This suggests a potential, yet unexplored, role for specific USPs, particularly those containing such motifs, in regulating *E.ulmoides* rubber biosynthesis in response to environmental cues. We hypothesize that *EuUSPs* gene containing this rubber particle motif, such as *EuUSP16*, regulate rubber synthesis under abiotic stress conditions.

Building upon the prior cloning of *EuUSP16* and the discovery of its rubber particle motif, this study aims to: systematically identify and characterize the *EuUSPs* gene family within *E.ulmoides*; analyze changes in *E.ulmoides* rubber content under adverse environmental conditions; and investigate the specific role of *EuUSP16* in *E.ulmoides* rubber synthesis and elucidate its underlying regulatory mechanisms. This work provides a crucial foundation for understanding the functional role of USPs in stress-induced rubber biosynthesis in *E.ulmoides*.

## 2 Materials and methods

### 2.1 Plant materials, growing conditions and treatment methods

*E.ulmoides* seedlings were cultivated in the following conditions: 16h light/8h dark photoperiod at 25°C and 70% relative humidity. Leaves, roots, and stem segments were collected from three-month-old *E.ulmoides* seedlings. Additionally, 15-month-old *E.ulmoides* seedlings subjected to natural drought or low-temperature stress for 10 days were sampled, with untreated 15-month-old seedlings serving as controls. All the samples were frozen in liquid nitrogen after collection and stored at −80°C until RNA isolation. Total RNA was extracted using Plant RNA Extraction Kit. All samples were processed in three biological replicates.

### 2.2 Analysis of the *EuUSPs* gene family

The genomic annotation data of *E.ulmoides* (Accession Number: CNA0148166), previously assembled by our research group from Guizhou Province, China, was downloaded from the CNGBdb database (https://db.cngb.org/). Concurrently, 42 A*.thaliana* USP gene and protein sequences were retrieved from the Arabidopsis Information Resource (TAIR) and UniProt databases. Putative *E.ulmoides* USP sequences were identified through local BLAST searches against the *E.ulmoides* genome using the *A.thaliana* sequences as queries. Additionally, protein sequences containing the USP (PF00582) domain were searched using HMMER 3.0 based on the InterPro-derived Hidden Markov Model (HMM) profile. Candidate *E.ulmoides* USP proteins obtained from these methods were combined into a non-redundant set. The conserved domains of these candidates were further validated using the NCBI Conserved Domain Database (CDD) and SMART. This comprehensive analysis ultimately identified the members of the *E.ulmoides* USP (EuUSPs) gene family. Genome-wide USP family sequences for *A.thaliana*, *Oryza sativa* (rice), *H.brasiliensis* (rubber tree), and *Taraxacum kok-saghyz* (rubber dandelion) were acquired from the Ensembl Plants database (http://plants.ensembl.org/index.html). A phylogenetic tree was constructed using the Neighbor-Joining (NJ) method in MEGA 12.0 software, based on the alignment of corresponding amino acid sequences performed with ClustalW (1000 bootstrap replicates). The resulting phylogenetic tree was visualized and refined using the iTOL web platform (https://itol.embl.de/). Subcellular localization of the EuUSPs proteins was predicted using WoLF PSORT, CELLO, and Plant-mPLoc. Conserved motifs were identified using the online MEME suite. Genomic BLAST alignments between *E.ulmoides*, *A.thaliana*, and *H.brasiliensis* were performed using TBtools to generate synteny and tandem duplication files, which were used to plot syntenic gene relationships. Gene Ontology (GO) analysis for the *EuUSPs* genes was conducted using eggNOG-mapper.

### 2.3 The influence of environmental treatments on the expression levels of *EuUSPs* genes

qRT-PCR primers for 11 genes in the *EuUSPs* gene family were designed using Premier 5.0 and Primer-BLAST (**Supplementary Table 1**) and synthesized by Sangon Biotech. qRT-PCR was performed using SYBR GreenI dye-based qPCR premix. The reaction mixture (10 μL) consisted of: 5 μL of 2× Universal Blue SYBR Green qPCR Master Mix, 1 μL of cDNA, 0.2 μL each of forward and reverse primers (10 μmol·L^-^¹), and 3.6 μL of ddH_2_O. The reaction program was as follows: initial denaturation at 95°C for 30 s; followed by 40 cycles of denaturation at 95°C for 15 s, annealing at 60°C for 30 s, and extension at 72°C for 30 s. Relative expression levels were calculated using the 2^−ΔΔCt^ method (Livak and Schmittgen, 2001), with the *EuActin* gene as the internal reference. At least three independent biological replicates and three technical replicates were performed for each sample.

### 2.4 Functional analysis of *EuUSP16* gene in *E.ulmoides* rubber synthesis

The previously constructed pSH737-*EuUSP16* overexpression vector and Virus-Induced Gene Silencing (VIGS) vector (pTRV2-*EuUSP16*) were genetically transformed into *E.ulmoides* via Agrobacterium-mediated transformation. The expression levels of *E.ulmoides* rubber biosynthesis-related genes and the rubber content were then analyzed. The primer sequences for the *E.ulmoides* rubber biosynthesis-related genes are listed in **Supplementary Table 1**, and the reaction system was the same as described in section 2.3.

### 2.5 *E.ulmoides* rubber content determination

Extraction of *E.ulmoides* rubber was performed with modifications based on the methods described by Lu et al. (2004) and Xie et al. (2013): Leaves and stem segments (approximately 10 g) of *E.ulmoides* after 10 days of drought and low-temperature treatments respectively were placed in an oven at 120°C for enzyme deactivation (15 min). After drying, the sample mass was recorded. The samples were ground into powder, mixed with 10% NaOH solution (200 mL), and subjected to a 3-hour water bath at 100°C, followed by 20 min of high-temperature/high-pressure treatment at 120°C. Residual NaOH solution was removed by rinsing under running water. The samples were then transferred to bottles containing 200 mL of purified water and incubated in a 60°C water bath for 3 h, followed by drying in an oven at 65°C. Petroleum ether (boiling range: 60-90°C) was added to a Soxhlet extractor at a solid-to-liquid ratio of 1:15 for 20 h of extraction. The petroleum ether solution was decanted into pre-weighed 50 mL centrifuge tubes and evaporated in a 37°C oven. Anhydrous ethanol was added to wash the precipitate repeatedly until white refined rubber was obtained. After oven-drying, the mass of the refined rubber was recorded, and the rubber content was calculated using the formula: Rubber content (%) = (Mass of refined rubber/Sample mass)×100%. Three biological replicates for all experiments.

### 2.6 Cloning and sequence analysis of *EuUSP16* promoter

Specific primers were designed to amplify *EuUSP16* promoter sequences of different lengths, and the cloned promoter fragments were constructed with the pCAMBIA1381Z vector. The constructed vectors were subsequently sent to BGI for sequencing. The primers used are shown in **Supplementary Table 1**.

### 2.7 Construction of the plant transformation vector and genetic transformation of tobacco

*EuUSP16* promoter DNA fragments of different lengths(1967 bp/1286 bp/1193 bp/273 bp) were connected to the *Eco*RI and *Bam*HI enzyme restriction sites of the pCAMBIA1381Z vector to obtain the following four vectors: P_EuUSP16-1,_P_EuUSP16-2_, P_EuUSP16-3_,P_EuUSP16-4_. These vectors were used to transform Agrobacterium GV3101, which was subsequently genetically transformed into tobacco. PCR was used to identify the transgenic tobacco plants for further study. The primers used are shown in **Supplementary Table 1**.

### 2.8 Yeast one-hybrid pairwise validation

The prey vector pGADT7 was double-digested with restriction enzymes *Bam*HI and *Eco*RI. The coding sequence (CDS) of *EuDof* was then cloned into the linearized pGADT7 vector using homologous recombination. This generated the pGADT7-EuDof prey construct for subsequent pairwise yeast one-hybrid validation of the interaction between pGADT7-EuDof and pHIS2-P_EuUSP16_.

### 2.9 Dual-luciferase reporter assay

The reporter vector pGreenII 62-SK and the effector vector pGreenII 0800-LUC were double-digested with *Bam*HI and *Kpn*I, respectively. The CDS of *EuDOf* was cloned into the linearized pGreenII 62-SK vector, while the P_EuUSP16–1_ promoter fragment was cloned into the linearized pGreenII 0800-LUC vector, using homologous recombination. Plasmid DNA from sequence-verified clones was extracted and transformed into Agrobacterium tumefaciens GV3101 (pSoup) competent cells. The bacterial suspensions were infiltrated into young, fully expanded leaves of 3–4-week-old tobacco plants using a needleless syringe. Infiltrated plants were maintained under standard growth conditions for 48 h. Luminescence was then visualized and quantified using a plant *in vivo* imaging system.

### 2.10 Construction of the pSH737-*EuDof* overexpression vector and genetic transformation of P_EuUSP16–1_ transgenic tobacco

The pSH737 overexpression vector plasmid was double-digested with *Xba*I and *Eco*RI. The CDS of the *EuDof* gene was cloned into the plant overexpression vector pSH737 using homologous recombination. Transgenic tobacco line P_EuUSP16–1_ was used as the starting material. After genetic transformation of tobacco, the expression level of the β-glucuronidase (GUS) gene was assessed.

### 2.11 Statistical analysis

Statistical analysis and graphing were performed using Excel 2010 and GraphPad Prism 9.5 software. Data differences were assessed by one-way ANOVA, with statistical significance denoted as follows: different lowercase letters indicate significant differences (*P<0.05*), while asterisks represent *P<0.05, **P<0.01, ***P<0.001, and**P<0.0001.

## 3 Results

### 3.1 Identification of the *EuUSPs* gene family and screening of genes associated with *E.ulmoides* rubber biosynthesis

**Figure 1:**
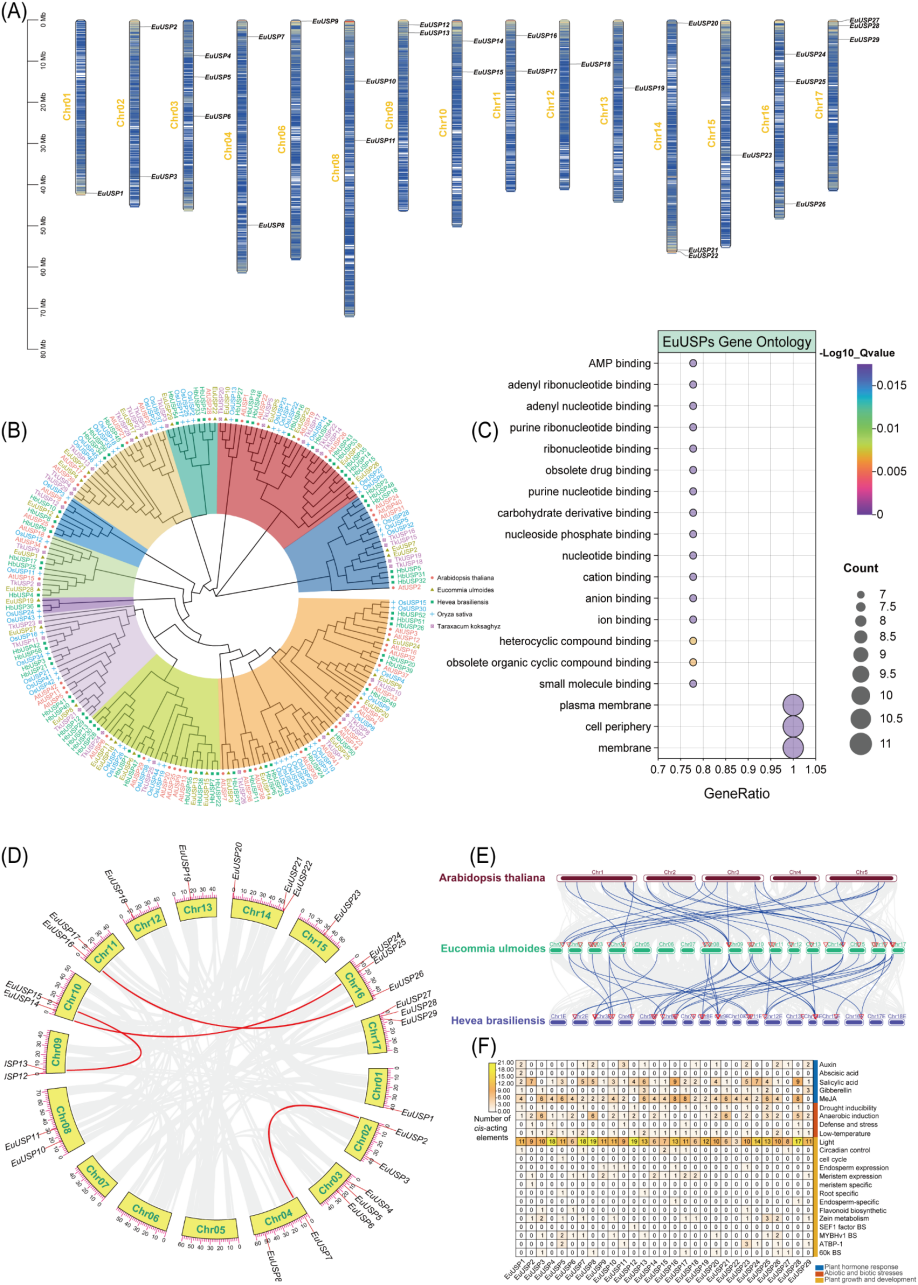
Identification of *EuUSPs* genes in *E.ulmoides.* **(A)** Chromosomal mapping. **(B)** Phylogenetic relationship among the EuUSPs proteins of the 5 plant species. **(C)** Gene ontology analysis. **(D)** Collinearity analysis of *EuUSPs* gene family in *E. ulmoides*. **(E)** Collinearity relation of USP genes in the genomes of *A.thaliana* and *H.brasiliensis*. **(F)** Analysis on *cis*-acting elements.

A total of 29 *EuUSPs* genes were identified and named *EuUSP1* to *EuUSP29* according to their chromosomal positions (**Figure 1A**). The physicochemical properties of the encoded proteins are shown in **Supplementary Table 2**. The 29 EuUSPs proteins contain 99 to 892 amino acid residues with relative molecular masses ranging from 11.28 to 98.86 kDa and theoretical isoelectric points (pI) between 4.81 and 10.37. These results indicate substantial variation in protein size and molecular weight within the EuUSPs family, suggesting diverse functional domains and structural features. The instability index ranged from 24.1 to 68.26, while the aliphatic index varied between 77.01 and 104.55 (23 proteins>80). This suggests moderate to high thermostability in most EuUSPs proteins. Twenty- five EuUSPs proteins exhibited negative grand average of hydropathicity values, indicating predominantly hydrophilic characteristics. Subcellular localization predictions revealed primary localization in the cytoplasm, chloroplasts, and mitochondria, with secondary localization in the nucleus, golgi apparatus, and endoplasmic reticulum.

Phylogenetic analysis was conducted between 29 *EuUSPs* gene family members from *E.ulmoides* and USP genes from four representative species: *A.thaliana* (42 genes), *H.brasiliensis* (58 genes), *Oryza sativa* (46 genes), and *T.kok-saghyz* (29 genes). The resulting phylogenetic tree is shown in **Figure 1B**. The 204 USP protein sequences clustered into nine subgroups (I-IX), containing 50, 49, 3, 12, 8, 32, 5, 26, and 19 members respectively. Phylogenetic tree analysis revealed that EuUSP5, EuUSP16, EuUSP23, and EuUSP26 (all containing rubber particle protein peptides; unpublished data) consistently clustered with USPs from latex-producing plants (either *H.brasiliensis* or *T.kok-saghyz*). This suggests that EuUSPs harboring rubber particle peptides share high homology with those from laticiferous plants (*H.brasiliensis* and *T.kok-saghyz*), potentially indicating functional conservation in *E.ulmoides* rubber biosynthesis. Analysis of conserved motifs in EuUSPs proteins revealed that 27 members (all except EuUSP20 and EuUSP29) contain Motif2 and Motif8. Notably, Motif9 was exclusively present in EuUSP5,16,23, and 26. These distinctive motif architectures provide the theoretical foundation for gene classification and functional prediction. All 29 EuUSPs proteins contain the USP domain. Gene structure analysis demonstrated: the 29 *EuUSPs* genes contained 0–10 introns, with only *EuUSP7* being intron-free (**Supplementary Figure 1**). To investigate the collinear relationships between *EuUSPs* and *USP* genes from other species, this study analyzed syntenic gene pairs among *E.ulmoides*, *A.thaliana*, and *H.brasiliensis*. As shown in **Figure 1E**, 17 collinear gene pairs were identified between *EuUSPs* and *AtUSPs*, while 21 collinear pairs existed between *EuUSPs* and *HbUSPs*. The higher frequency of syntenic events with the rubber-producing plant compared to the non-laticiferous species suggests evolutionary conservation of these USP genes within the *EuUSPs* family. This collinearity advantage reflects both the phylogenetic proximity between *E. ulmoides* and rubber tree, and underscores the potential functional importance of these genes in rubber biosynthesis. Analysis of duplication events in *EuUSPs* genes revealed that collinearity primarily occurs on chromosomes 2, 4, 9, 10, 11, and 16 (**Figure 1D**). Four segmental duplication events were identified within the *EuUSPs* family, involving 8 genes forming 4 duplicated pairs: *EuUSP2*-*EuUSP7*, *EuUSP12*-*EuUSP15*, *EuUSP14*-*EuUSP25*, and *EuUSP16*-*EuUSP26*. These gene pairs represent collinear genes within syntenic blocks. While *EuUSP14* and *EuUSP15* exhibit adjacent duplication in close chromosomal proximity, no evidence of tandem duplication events was detected among *EuUSPs* genes. Analysis of *cis*-regulatory elements in *EuUSPs* gene family promoters is presented in **Figure 1F**. Promoter regions of *E.ulmoides EuUSPs* are enriched with diverse regulatory elements. Major *cis*-elements include phytohormone-responsive motifs: abscisic acid (ABA), salicylic acid (SA), auxin (IAA), methyl jasmonate(MeJA), and gibberellin response elements. Four types of stress-related *cis*-elements were identified: anaerobic induction, low-temperature response, defense/stress response, and drought-inducible elements. Additionally, promoters contain elements associated with cell cycle regulation, circadian rhythm, meristem expression, and flavonoid biosynthesis. GO analysis was performed on *EuUSPs* genes, with the results shown in **Figure 1C**. Molecular Function: Eleven *EuUSPs* genes (*EuUSP1*, *EuUSP2*, *EuUSP5*, *EuUSP7*, *EuUSP8*, *EuUSP10*, *EuUSP12*, *EuUSP19*, *EuUSP22*, *EuUSP27*, and *EuUSP29*) showed significant enrichment in molecular functions. These include:AMP binding (GO:0016208), Adenyl ribonucleotide binding (GO:0032559), Adenyl nucleotide binding (GO:0030554) and other terms. Four genes (*EuUSP2*, *EuUSP7*, *EuUSP8*, and *EuUSP27*) exhibited significant enrichment in cellular components including:Plasma membrane (GO:0005886), cell periphery (GO:0071944) and membrane (GO:0016020). These findings indicate that *EuUSPs* genes participate in energy metabolism, signal transduction, and molecular recognition processes. No significant enrichment was observed for any Biological Process terms.

### 3.2 Tissue-specific expression analysis of *EuUSPs*

**Figure 2:**
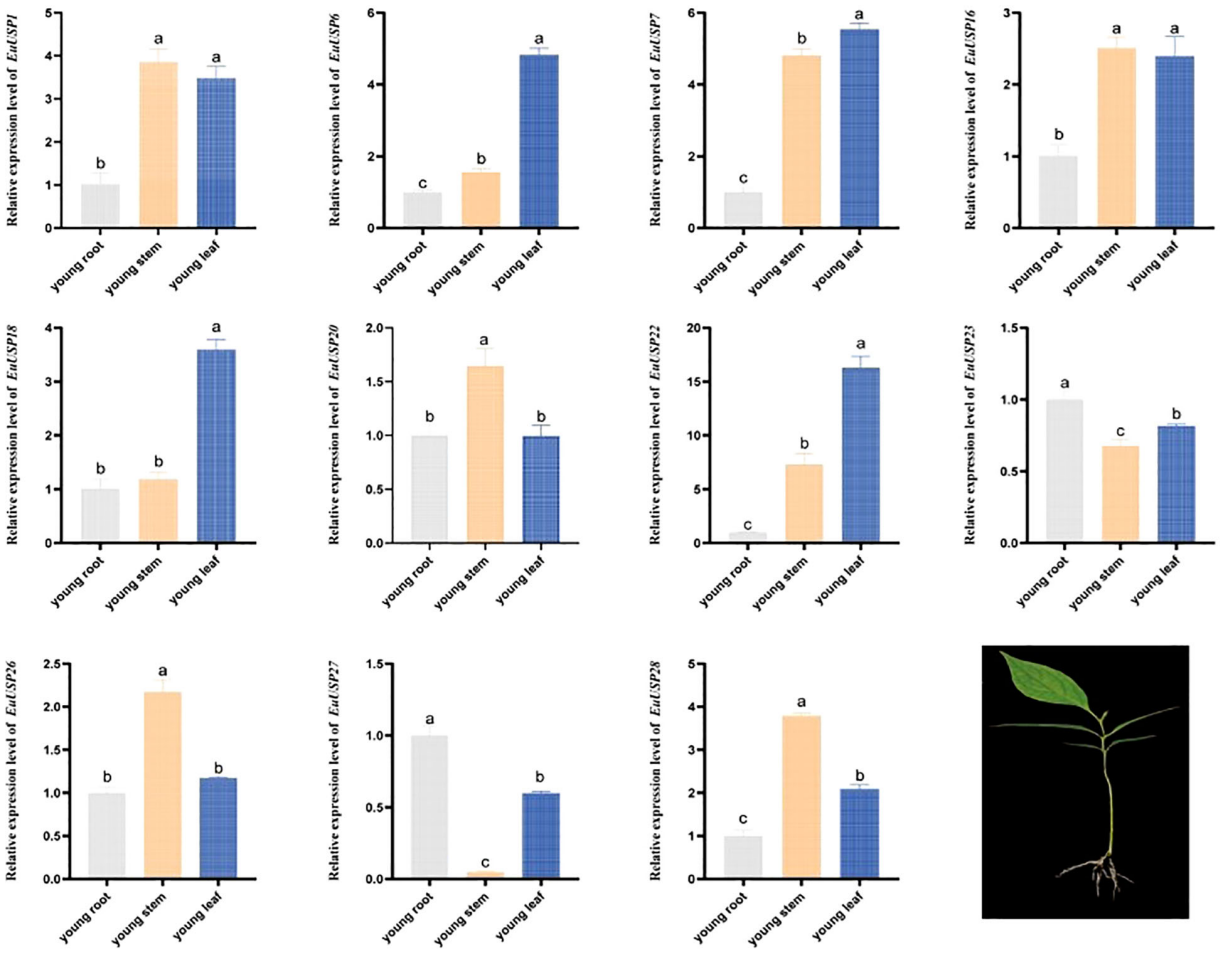
Tissue-specific expression pattern of the *EuUSPs* gene in *E.ulmoides.* Different lowercase letters indicate significant differences (*P<0.05*).

To validate the expression patterns of *EuUSPs* genes in the roots, stems, and leaves of *E.ulmoides* seedlings, we analyzed their expression in three-month-old seedlings. The results are presented in **Figure 2**. *EuUSP1*, *EuUSP16*, *EuUSP20*, *EuUSP26*, and *EuUSP28* are highly expressed primarily in stems with high rubber content.

### 3.3 Effects of drought and low-temperature treatments on *EuUSPs* gene expression and rubber content in *E.ulmoides* seedlings

**Figure 3:**
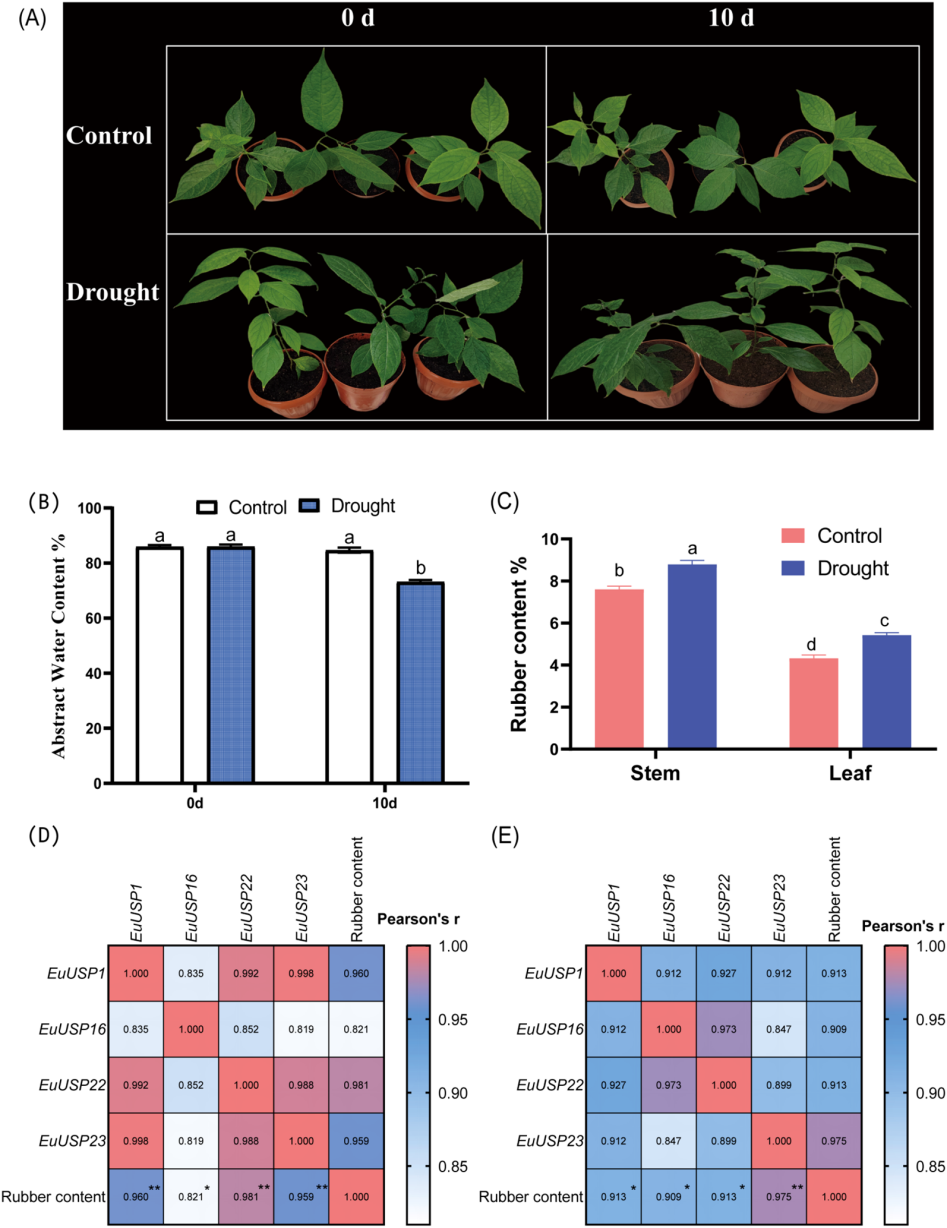
Pearson correlation analysis between *EuUSPs* gene expression and rubber content under 10-day drought stress. **(A)** Drought-treated *E.ulmoides* seedlings. **(B)** The absolute water content in *E*. *ulmoides* leaves before and after drought treatment. **(C)** Rubber content. **(D)** Correlation between *EuUSPs* expression in stems and rubber content. **(E)** Correlation between *EuUSPs* expression in leaves and rubber content. Control: negative control. Different lowercase letters indicate significant differences (*P<0.05*). *P<0.05, **P<0.01, ***P<0.001, ****P<0.0001.

**Figure 4:**
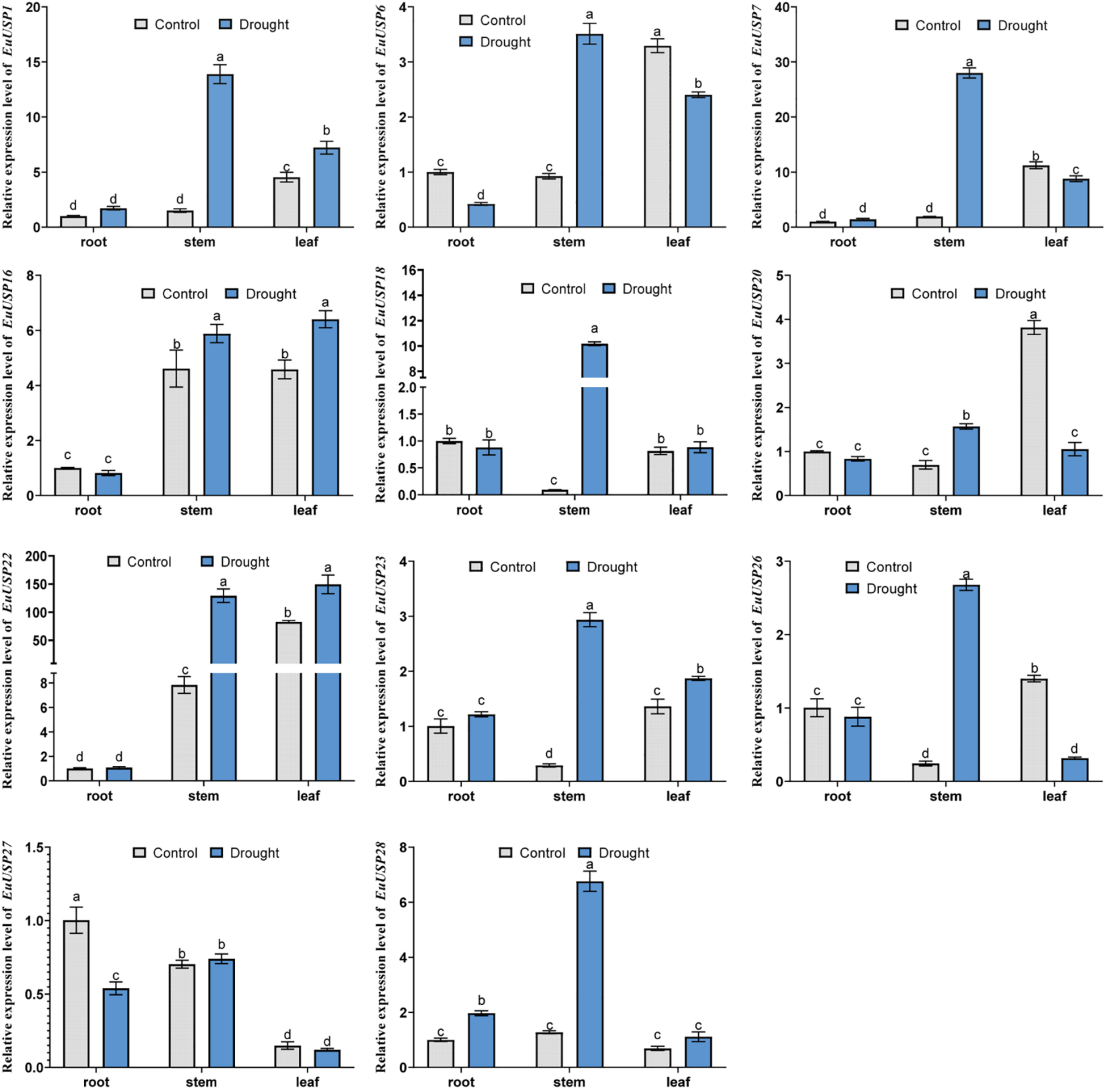
The expression analysis of *EuUSPs* gene in *E.ulmoides* after drought treatment. Control: negative control. different lowercase letters indicate significant differences (*P<0.05*).

After 10 days of drought treatment in 15-month-old E.ulmoides seedlings, phenotypic changes are shown in **Figure 3A**. The absolute water content was 84.592% in the control group versus 73.191% in the drought-treated group, showing a significant decrease compared to the control (**Figure 3B**). Expression levels of *EuUSPs* in roots, stems, and leaves and rubber content were analyzed. *EuUSP1*, *EuUSP6*, *EuUSP7*, *EuUSP16*, *EuUSP18*, *EuUSP20*, *EuUSP22*, *EuUSP23*, *EuUSP26*, and *EuUSP28* exhibited significant upregulation (2.28-to 108.19-fold increase) in stems compared to the control. *EuUSP1*, *EuUSP16*, *EuUSP22*, and *EuUSP23* showed significant upregulation (1.38- to 1.80-fold increase) in leaves (**Figure 4**). Measurement of rubber content in *E.ulmoides* stems and leaves revealed the following: In control samples, stem and leaf rubber contents were 7.605% and 4.326%, respectively. In the experimental group, stem and leaf rubber contents were 8.989% and 5.426%, respectively. Rubber content in both stems and leaves was significantly higher than in the control group (**Figure 3C**). *EuUSP1*, *EuUSP16*, *EuUSP22*, and *EuUSP23* upregulation in stems/leaves positively correlated with increased laticifer content (*P<0.05*) (**Figures 3D, E**). These results indicate that expression of *EuUSP1*, *EuUSP16*, *EuUSP22*, and *EuUSP23* influences rubber biosynthesis in stems and leaves.

**Figure 5:**
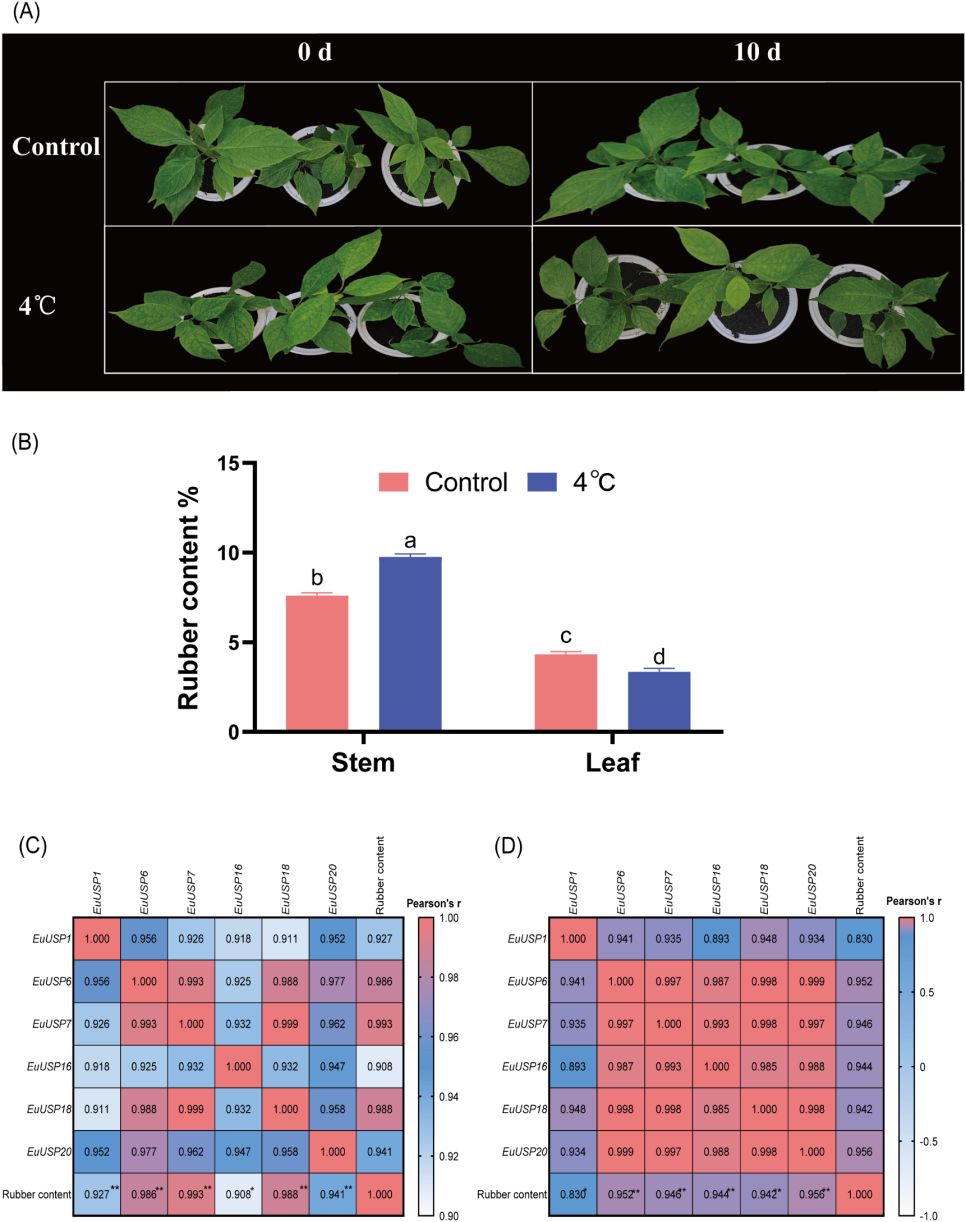
Pearson correlation between *EuUSPs* gene expression and rubber content under 4°C treatment. **(A)** 4°C-treated *E.ulmoides* seedlings **(B)** Rubber content. **(C)** Correlation between *EuUSPs* expression in stems and rubber content. **(D)** Correlation between *EuUSPs* expression in leaves and rubber content. Note: Control, negative control. Different lowercase letters indicate significant differences (*P<0.05*). *P<0.05, **P<0.01, ***P<0.001, ****P<0.0001..

**Figure 6:**
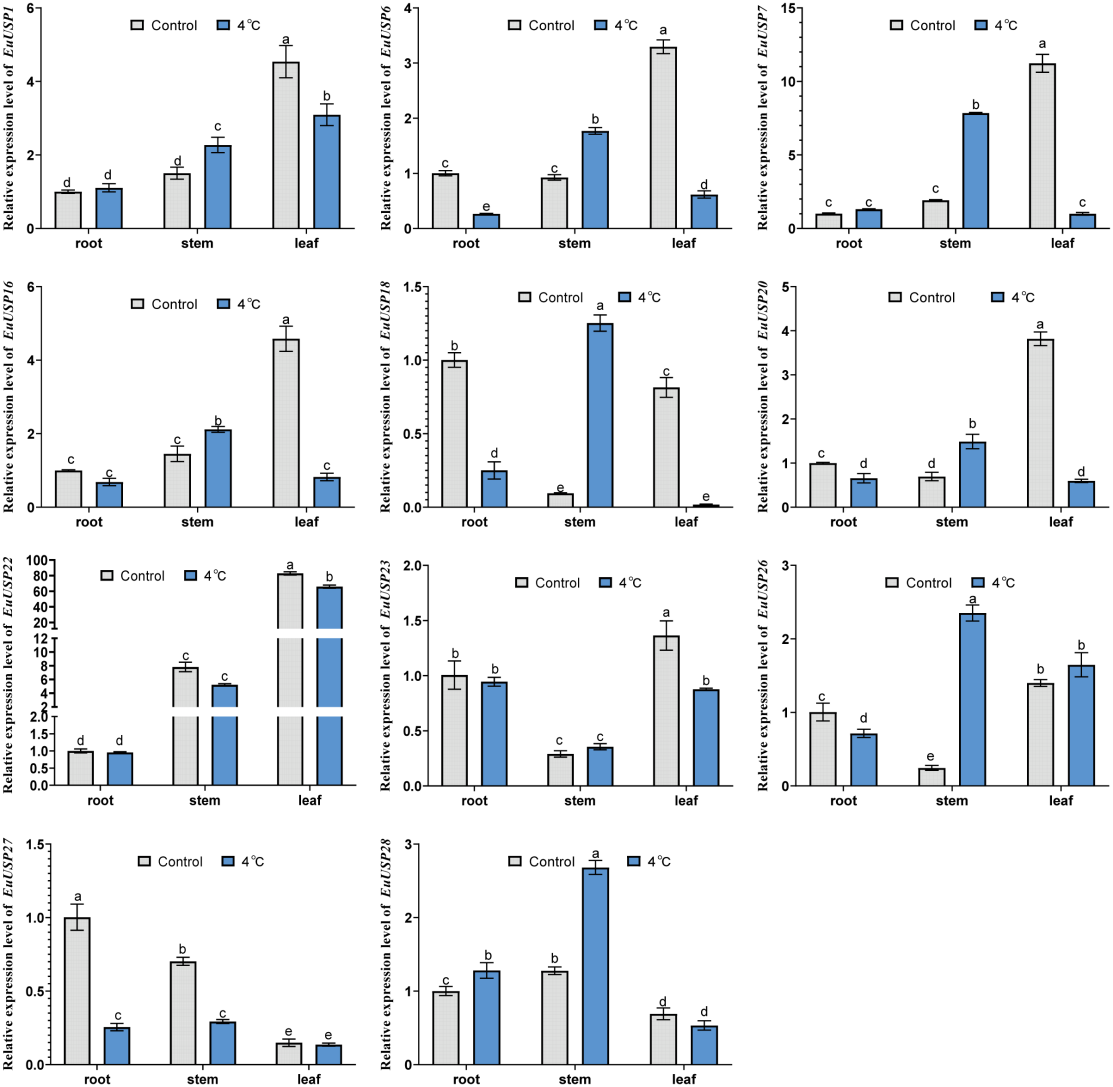
The expression analysis of *EuUSPs* gene in after 4°C treatment. Control: 25°C treatment. Different lowercase letters indicate significant differences (*P<0.05*).

Analysis of *EuUSPs* gene expression and rubber content was conducted after 10 days of low-temperature treatment (4°C) in 15-month-old seedlings (**Figure 5A**). The results showed that in stems, the relative expression levels of *EuUSP1*, *EuUSP6*, *EuUSP7*, *EuUSP16*, *EuUSP18*, *EuUSP20*, *EuUSP26*, and *EuUSP28* increased by 1.46- to 13.29-fold compared to the control group. In leaves, the expression levels of *EuUSP1*, *EuUSP6*, *EuUSP7*, *EuUSP16*, *EuUSP18*, *EuUSP20*, *EuUSP22*, and *EuUSP23* significantly decreased to 0.089-0.79 times that of the control group (**Figure 6**). Measurement of rubber content in *E.ulmoides* stems and leaves revealed: Control group: Stem content 7.605%, leaf content 4.326%. Low-temperature treatment group: Stem content 9.764%, leaf content 3.349%. After 10 days of 4°C treatment, stem rubber content significantly increased while leaf content significantly decreased compared to controls (**Figure 5B**). Additionally, expression levels of six genes (*EuUSP1*, *EuUSP6*, *EuUSP7*, *EuUSP16*, *EuUSP18*, and *EuUSP20*) in stems and leaves positively correlated with changes in rubber content (*P<0.05*) (**Figures 5C, D**). This indicates that expression of these six *EuUSPs* genes influences *E.ulmoides* rubber biosynthesis.

Under low-temperature and drought stress treatments, the expression levels of multiple *EuUSPs* genes were altered. Notably, *EuUSP16* expression consistently exhibited a positive correlation with rubber content. Furthermore, this gene encodes a protein containing a rubber particle protein motif. We therefore propose that *EuUSP16* plays a significant role in *E.ulmoides* rubber biosynthesis under stress conditions. Consequently, *EuUSP16* was selected for in-depth functional characterization.

### 3.4 The role of *EuUSP16* in the *E.ulmoides* rubber synthesis

**Figure 7:**
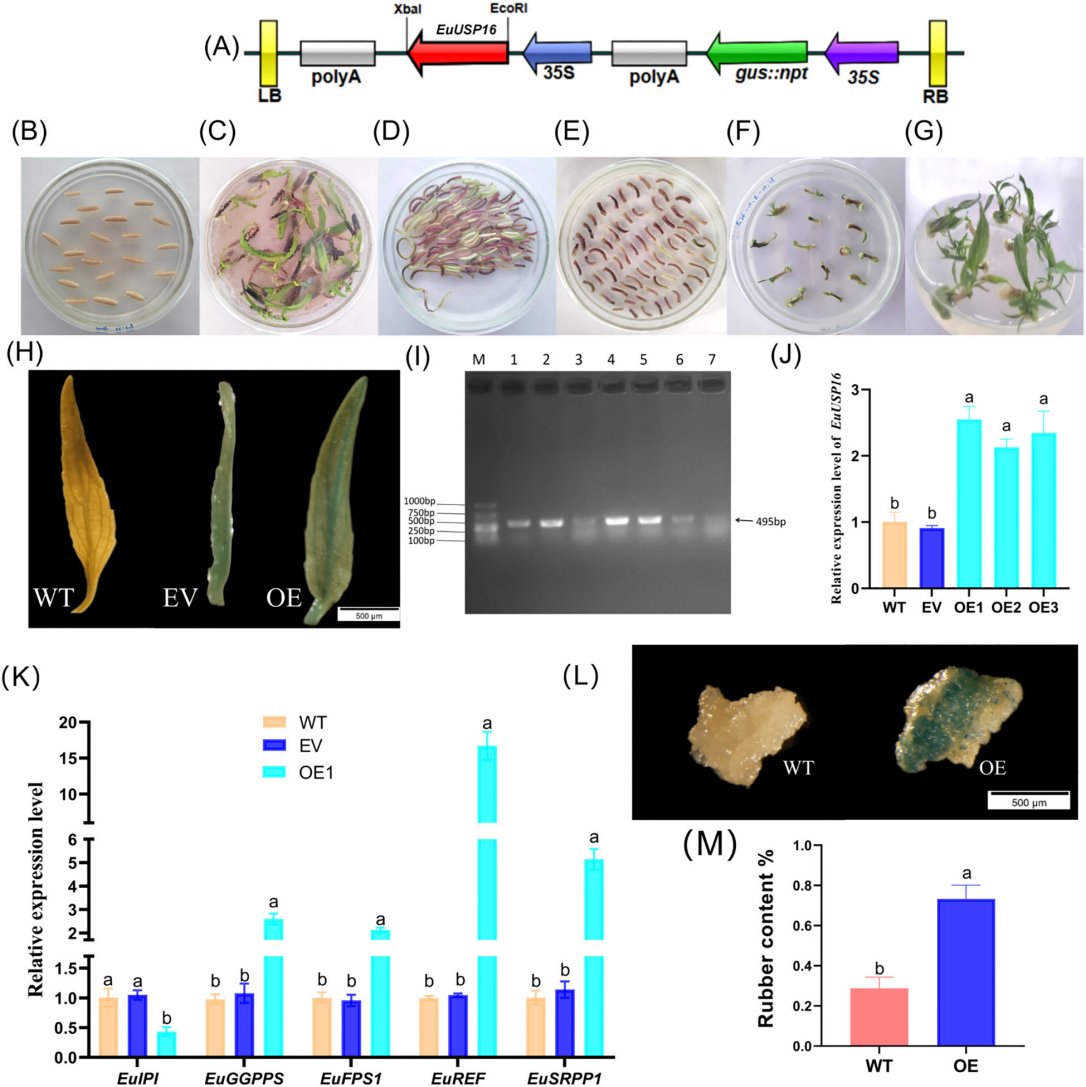
Functional analysis of the *EuUSP16* gene. **(A)** T-DNA structure of the pSH737-35S-*EuUSP16* vector. **(B-C)** Germination process of *E.ulmoides* seeds. **(D)** Co-cultivation. **(E)** Screening of resistant tissues. **(F-G)** Induction of resistant buds **(H)** GUS staining identification. **(I)** PCR verification of transgenic *EuUSP16* plants. **(J)** Gene expression levels in transgenic *EuUSP16* buds. **(K)** Expression of rubber synthesis-related genes in *EuUSP16*-overexpressing buds. **(L)** GUS staining of callus. **(M)** *E.ulmoides* rubber content in callus. Note: M, DL2000 marker; Different lowercase letters indicate significant differences at *P<0.05*.

The pSH737-35S-*EuUSP16* was genetically transformed into *E.ulmoides* hypocotyls (**Figures 7 A–G**). Six transgenic *EuUSP16* adventitious buds of *E.ulmoides* were obtained following GUS staining and PCR verification, as shown in **Figures 7G, H**. In the transgenic *EuUSP16* buds, the relative expression levels of *EuGGPPS*, *EuFPS1*, *EuREF*, and *EuSRPP1* genes were significantly higher than those in wild type (WT) and transgenic empty vector(EV) adventitious buds, measuring 2.60-, 2.12-, 16.71-, and 4.51-fold of WT, respectively, while the relative expression of *EuIPI* was significantly reduced to 0.43-fold of WT (**Figures 7J, K**). *E.ulmoides* rubber was extracted from (WT) and Over-Expression (OE) callus, with results presented in **Figure 7M**. The rubber content in WT callus was 0.288%, whereas that in EuUSP16-overexpressing callus was 0.733%, indicating a significant increase. These findings demonstrate that *EuUSP16* overexpression enhances the expression of key *E.ulmoides* rubber synthesis genes such as *EuFPS1*, further promoting *E.ulmoides* rubber synthesis.

**Figure 8:**
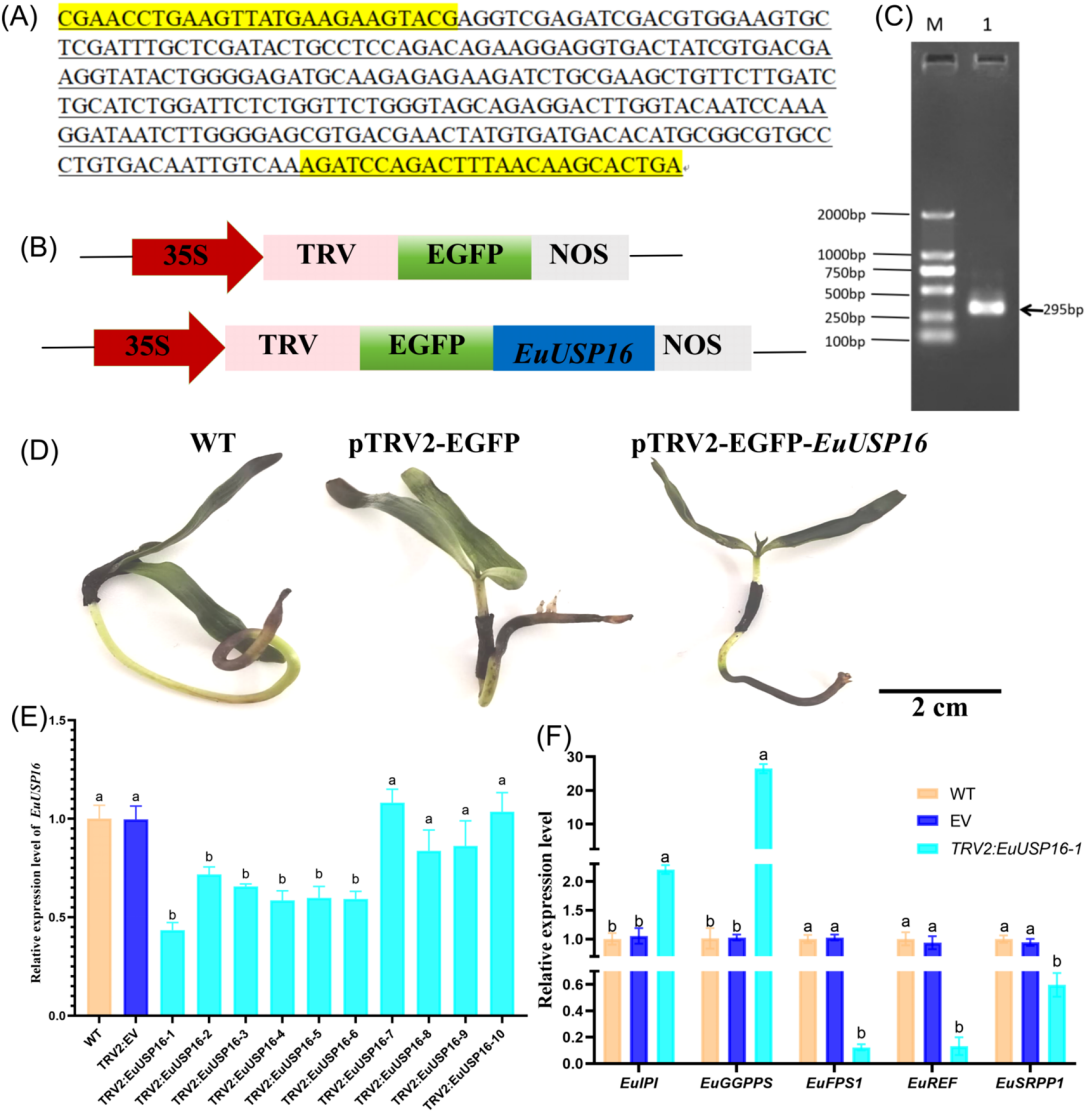
Effects of *EuUSP16* gene silencing on the expression of rubber biosynthesis-related genes in *E.ulmoides*. **(A)** Virus-Induced Gene Silencing (VIGS) fragment. **(B)** Schematic diagram of pTRV2-GFP vector. **(C)** PCR detection results. M: DL 2000 DNA Marker Lane 1:pTRV2-GFP-*EuUSP16* plasmid. **(D)** Phenotype of *EuUSP16*-silenced *E.ulmoides* seedlings. **(E)** Gene expression levels in *EuUSP16*-silenced *E.ulmoides.* **(F)** Expression levels of rubber biosynthesis-related genes in *EuUSP16*-silenced *E.ulmoides.* Different lowercase letters indicate significant differences (*P<0.05*).

The construction process of the EuUSP16 gene silencing vector is shown in **Figures 8A–C**. As shown in **Figures 8D-F**, following qRT-PCR validation, six *E.ulmoides* seedlings with reduced relative expression levels of the *EuUSP16* gene were obtained. The line with the lowest expression, namely pTRV2-*EuUSP16*-1, was selected to analyze the relative expression levels of genes related to *E.ulmoides* rubber synthesis: *EuGGPPS*, *EuFPS1*, *EuREF*, and *EuSRPP1*. The results showed that in the pTRV2-*EuUSP16–*1 plants, the relative expression levels of the *EuFPS1*, *EuREF*, and *EuSRPP1* genes were significantly reduced compared with WT and TRV2 plants, reaching 0.12-fold, 0.13-fold, and 0.59-fold that of WT, respectively. In contrast, the relative expression levels of the *EuGGPPS* and *EuIPI* genes were significantly increased, reaching 26.50-fold and 2.21-fold that of WT, respectively. This indicates that *EuUSP16* silencing affects *E.ulmoides* rubber synthesis by reducing the expression of key enzyme genes such as *EuFPS1*.

### 3.5 Effects of hormone and environmental treatments on the activity of the *EuUSP16* promoter

**Figure 9:**
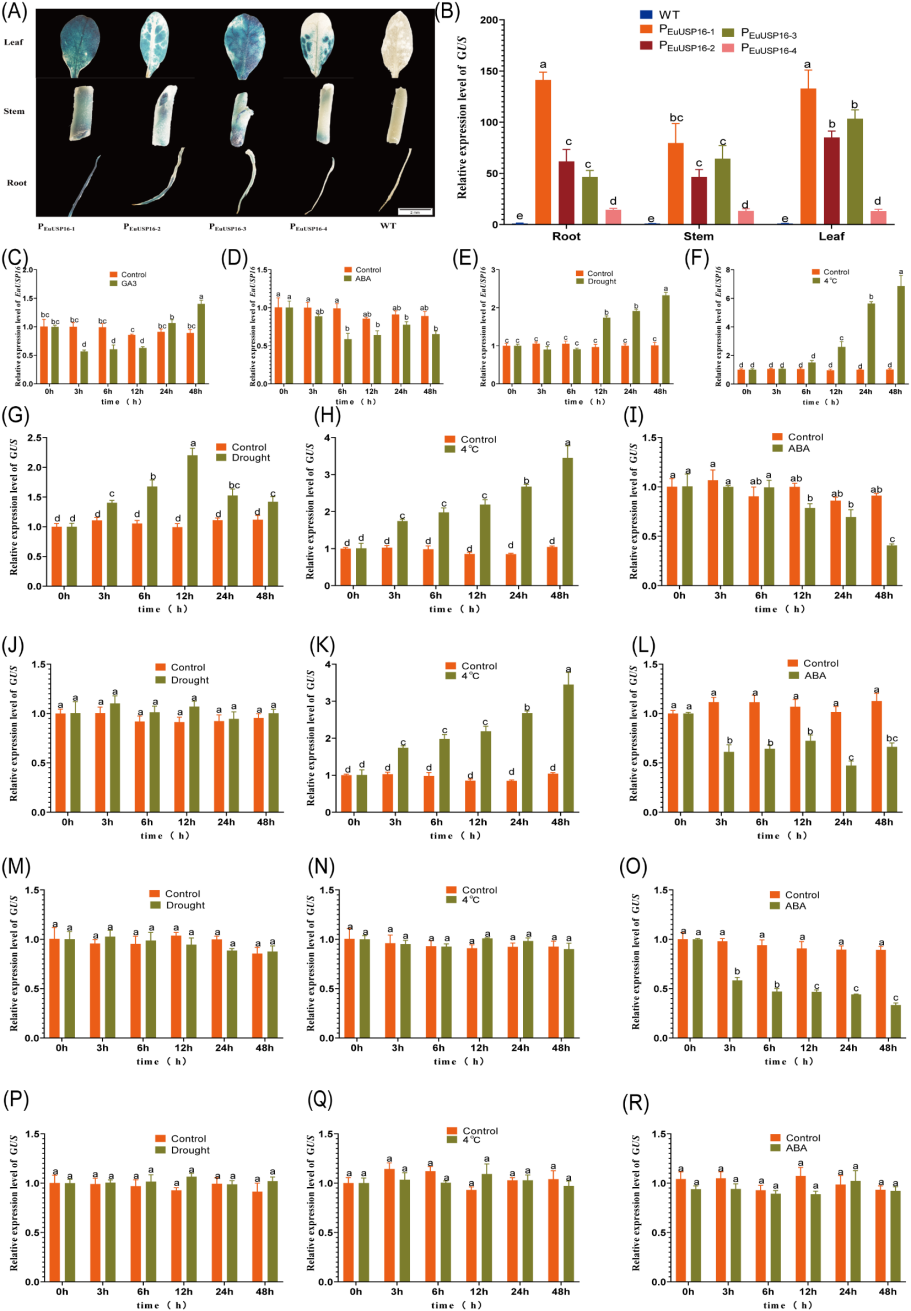
Effects of hormone and environmental treatments on the activity of the *EuUSP16* promoter. **(A)** GUS histochemical staining in roots, stems, and leaves of transgenic tobacco. **(B)** Relative expression levels of the *GUS* gene in transgenic tobacco harboring four *EuUSP16* promoter fragments. **(C-F)** Relative expression levels of *EuUSP16* in *E.ulmoides* seedlings treated with 100 μmol·L^-^¹GA_3_, 100 μmol·L^-^¹ ABA, drought, and low temperature, respectively. **(G-R)** Relative *GUS* expression in transgenic tobacco carrying four *EuUSP16* promoter fragments under treatments:**(G-I)**, P_EuUSP16–1_ transgenic tobacco treated with 300 mmol·L^-^¹ mannitol (simulated drought), 4°C, or 100 μmol·L^-^¹ ABA spray, **(J-L)** P_EuUSP16–2_ transgenic tobacco treated identically, **(M-O)** P_EuUSP16–3_ transgenic tobacco treated identically, **(P-R)** P_EuUSP16–4_ transgenic tobacco treated identically. Control, Untreated group; 0 h: Pre-treatment baseline; Different lowercase letters indicate significant differences (*P < 0.05*).

This study cloned a 1967 bp promoter sequence upstream of the ATG start codon of the *EuUSP16* gene. The positions of response elements were analyzed, and four plant expression vectors driving *GUS* gene expression were constructed: the full-length promoter P_EuUSP16-1_ (1967 bp), and three deletion constructs, P_EuUSP16-2_ (1286 bp) lacking the drought response element, P_EuUSP16-3_ (1193 bp) lacking the low-temperature response element, and P_EuUSP16-4_ (273 bp) lacking the ABA response element. Genetic transformation was performed in tobacco. *GUS* gene expression analysis revealed that the *EuUSP16* promoter functions as a constitutive promoter (**Figures 9A, B**). Transgenic tobacco harboring P_EuUSP16–1_ exhibited the highest GUS activity, while those with P_EuUSP16–4_ showed the lowest. No significant difference in *GUS* expression was observed between transgenic lines carrying P_EuUSP16–2_ and P_EuUSP16-3_, although both differed significantly from lines harboring either P_EuUSP16–1_ or P_EuUSP16-4_. These results indicate that the core promoter region of *EuUSP16* resides within the -273 bp to -1 bp fragment.

In P_EuUSP16–1_ transgenic tobacco plants subjected to drought stress, the relative *GUS* expression level progressively increased from 3 h to 12 h of treatment, reaching a 2.2-fold induction at 12 h (**Figure 9G**). Following 4°C treatment, the relative *GUS* expression in leaves of P_EuUSP16–1_ transgenic plants increased over time, peaking at 48 h with a 3.4-fold increase compared to the pre-treatment level (**Figure 9H**). Conversely, treatment with ABA solution significantly reduced *GUS* expression in P_EuUSP16–1_ transgenic leaves between 12 h and 48 h, reaching levels 0.4- to 0.78-fold of the control, indicating that ABA significantly suppresses *GUS* expression (**Figure 9I**). These findings demonstrate the presence of response elements associated with MBS, LTR, and ABRE within the P_EuUSP16–1_ promoter fragment. Drought treatment of P_EuUSP16–2_ transgenic plants resulted in no significant change in relative *GUS* expression (**Figure 9J**). However, 4°C treatment induced a significant increase in *GUS* expression, reaching 5.8-fold of the control at 48 h (**Figure 9K**). Spray treatment with ABA solution caused a significant decrease in *GUS* expression, with levels dropping to 0.42-fold of the control at 24 h (**Figure 9L**). These results indicate that the P_EuUSP16–2_ promoter fragment contains functional LTR and ABRE response elements. No significant alterations in relative *GUS* expression were observed in leaves of P_EuUSP16–3_ transgenic plants following either drought or 4°C treatment (**Figures 9M, N**). ABA treatment, however, led to a gradual decline in *GUS* expression in P_EuUSP16–3_ transgenic leaves, reaching a minimum of 0.34-fold of the control at 48 h (**Figures 9O**). This suggests the presence of an ABRE-related response element within P_EuUSP16-3_. Treatments with drought, 4°C, or ABA solution elicited no significant differences in relative *GUS* expression in leaves of P_EuUSP16–4_ transgenic plants (**Figures 9P-R**). This indicates the absence of functional MBS, LTR, and ABRE response elements within the P_EuUSP16–4_ promoter fragment.

In summary, the *EuUSP16* gene promoter harbors MBS (CAACTG), LTR (CCGAAA), and ABRE (ACGTG) response elements. *GUS* gene expression driven by this promoter can be induced by low temperature and drought stress, but is repressed by ABA treatment. This expression pattern is largely consistent with that of *EuUSP16* observed in *E.ulmoides seedlings* (**Figures 9C-F**).

### 3.6 The EuDof transcription factor controls *EuUSP16* gene expression through binding to its promoter

To identify upstream regulators of the *EuUSP16* gene, a yeast one-hybrid (Y1H) screening assay was performed. A total of 36 initial positive clones were screened (**Supplementary Table 3**). BLAST analysis revealed one clone encoding a transcription factor, specifically a DNA-binding with one finger (Dof) protein, which is a plant-specific transcription factor. The CDS of the *EuDof* gene was amplified by PCR, yielding a 1272 bp fragment encoding 423 amino acids. The encoded protein belongs to the zf-Dof superfamily; thus, the gene was designated *EuDof*.

**Figure 10:**
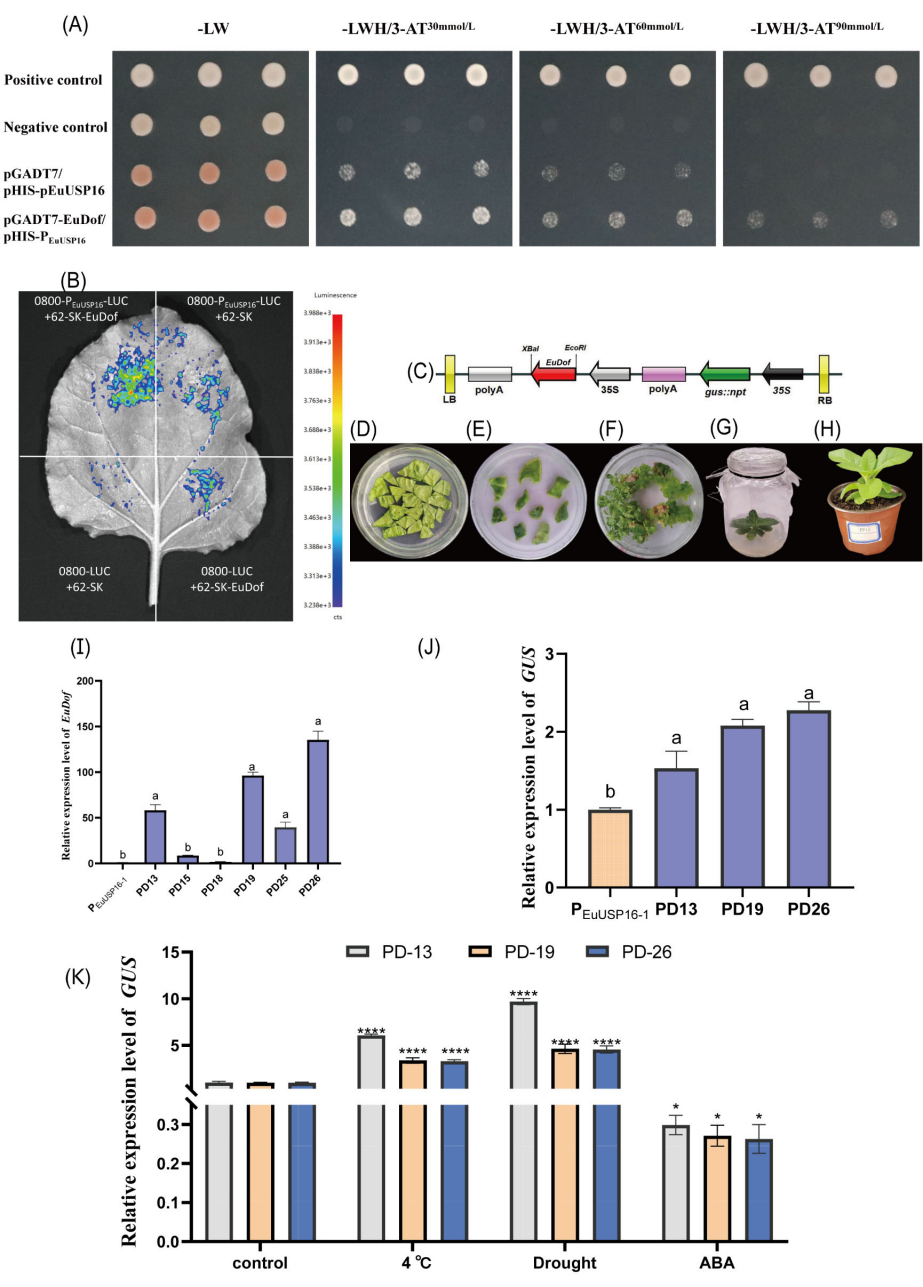
Interaction analysis between the *EuUSP16* promoter and EuDof transcription factor. **(A)** Y1H one-on-one validation of P_EuUSP16–1_ and EuDof interaction. **(B)** Dual-luciferase (LUC) reporter assay. Note for b: Negative controls: 0800-P_EuUSP16_-LUC + 62-SK empty vector, 0800-LUC empty vector + 62-SK empty vector, 0800-LUC empty vector + 62-SK-EuDof; Experimental group: 0800-P_EuUSP16_-LUC + 62-SK-EuDof **(C)** T-DNA structure of the pSH737-35S-EuDof overexpression vector. **(D)** Co-cultivation of Agrobacterium with tobacco leaf discs **(E)**. Selection of antibiotic-resistant callus. **(F)** Shoot induction from transgenic callus. **(G)** PCR identification of positive transgenic plants. **(H)** Potted transgenic seedlings. **(I)** Expression analysis of the EuDof transgene in PD transgenic tobacco lines. **(J)** *GUS* gene expression analysis in PD transgenic tobacco lines. **(K)** *GUS* expression in PD transgenic tobacco lines following 4°C, drought, or 100μmol·L^-^¹ABA treatments. Note: Different lowercase letters indicate significant differences (*P<0.05*). *P<0.05, **P<0.01, ***P<0.001, ****P<0.0001..

To determine whether the *EuUSP16* promoter interacts with EuDof, yeast one-on-one assays and dual-luciferase reporter assays confirmed that the EuDof transcription factor directly binds to the *EuUSP16* promoter and activates *EuUSP16* expression (**Figures 10A, B**). Concurrently, the *EuDof* gene was cloned into the pSH737 plant overexpression vector. Agrobacterium-mediated genetic transformation was used to introduce *EuDof* into transgenic tobacco harboring the P_EuUSP16–1_ promoter construct (transgenic lines designated PD) (**Figures 10C-I**). Analysis of relative *GUS* gene expression revealed a significant increase in *GUS* levels in P_EuUSP16–1_ transgenic tobacco overexpressing EuDof (**Figures 10J**), further supporting the interaction between the *EuUSP16* promoter and the EuDof transcription factor.

Additionally, transgenic lines PD13, PD19, and PD26 were subjected to low temperature (4°C), drought (simulated drought), or 100 µmol/L ABA treatment. Samples were collected at 48 h, and *GUS* expression was quantified (**Figure 10K**). Following low-temperature and drought treatments, *GUS* expression in all three lines was significantly higher than in untreated controls, reaching levels 3.30- to 6.06-fold and 4.57- to 9.69-fold of the control, respectively. Conversely, ABA spray treatment significantly reduced relative *GUS* expression to 0.26- to 0.29-fold of the control. These results indicate that low-temperature and drought stress promote the binding of the EuDof transcription factor to the *EuUSP16* promoter, while ABA treatment has the opposite effect.

## 4 Discussion

### 4.1 Characterization of the *EuUSPs* gene family

Plant USPs play important roles in responding to abiotic and biotic stresses (Nabi et al., 2024; Tkaczuk et al., 2013). This study identified 29 *EuUSPs* gene family members from the *E.ulmoides* genome. The *EuUSPs* genes are unevenly distributed across the 15 chromosomes of *E. ulmoides*, and their distribution shows no clustering phenomenon, similar to the chromosomal distribution of the *USP* gene family in potato (Qi et al., 2024; Wang et al., 2024). Among the *EuUSPs* family members, 25 are hydrophilic proteins, and their subcellular localization is primarily in the cytoplasm, chloroplasts, mitochondria, and nucleus, consistent with the localization reported for *USP* gene family members in other plants (Arabia et al., 2021).

A phylogenetic tree revealed that *EuUSP5*, *EuUSP16*, *EuUSP23*, and *EuUSP26* from *E.ulmoides* show close phylogenetic relationships with USPs from *H.brasiliensis* and *T.kok-saghyz*, and have been detected in rubber particles (unpublished data). This suggests that these *EuUSPs* members might share similar evolutionary trajectories with other rubber-producing plants like *H.brasiliensis* and *T.kok-saghyz*, and could potentially participate in the regulation of rubber biosynthesis. The number and composition of motifs vary among the *EuUSPs* family members. Except for EuUSP20 and EuUSP29, the other 27 EuUSPs all contain Motif 2 and Motif 8, indicating high conservation within the *EuUSPs* gene family during evolution. Among the *EuUSPs* family members, *EuUSP7* lacks introns, while the other 27 *EuUSPs* possess 1 to 10 introns. Over half of the members contain either 2 introns or 3 introns. Gilbert found that introns in ancient gene structures correlate with gene evolution (Gilbert and Glynias, 1993). In *Gossypium hirsutum*, the U and F subfamilies of the GST family appeared first, followed by the evolution of other subfamilies (Xu et al., 2017). In this study, *EuUSP7* (with no introns) and *EuUSP2*, *EuUSP6*, *EuUSP19*, *EuUSP26* (each with only one intron) may represent the earliest evolved genes. The other members, with varying numbers of introns, likely appeared later during gene evolution. Intron-derived motifs can enhance mRNA accumulation and post-transcriptional gene regulation through both splicing-dependent and splicing-independent mechanisms (Gallegos and Rose, 2019). Therefore, it is hypothesized that the other 28 intron-containing *EuUSPs* evolved different numbers of introns to regulate gene expression. However, the possibility that the intronless *EuUSP7* resulted from intron loss due to retrotransposition cannot be excluded and requires further analysis and validation.

Synteny can be used to predict homologous gene sequences during evolution. Synteny analysis at the whole-genome level revealed that all 21 *EuUSPs* genes analyzed show syntenic homology with *HbUSPs* genes from *H.brasiliensis*, indicating conservation of *EuUSPs* genes during the domestication of *E.ulmoides*. Within the *EuUSPs* family, four segmental duplication events involving eight *EuUSPs* genes were identified, but no tandem duplication events were found. Gene duplication, as a major driver of plant genome evolution, provides the material basis for gene family expansion through the generation of genetic redundancy (Lawton-Rauh, 2003). The newly duplicated genes in *E.ulmoides*, under natural selection, can acquire biological functions divergent from the original genes through functional mutations (Conant and Wolfe, 2008). Notably, the functional diversity arising from such duplication events profoundly influences the dynamics of gene expression regulatory networks. This is manifested as spatio-temporal adjustments in mRNA expression levels and adaptive changes in the physicochemical properties of proteins (Zhu et al., 2022). Such multi-layered molecular innovation mechanisms ultimately shape the evolutionary plasticity of plants in responding to environmental changes.

### 4.2 Expression characteristics and functional role of *EuUSPs* genes in *E.ulmoides* rubber biosynthesis

We found that the promoter regions of the *EuUSPs* gene family in *E.ulmoides* are rich in regulatory elements, primarily including plant hormone response elements. Additionally, four types of *cis*-acting elements related to abiotic and biotic stresses were identified, involving plant responses to abiotic stresses such as anaerobic conditions, low temperature, defense stress, and drought. Studies have shown that the environment can affect the content of plant secondary metabolites (Copolovici et al., 2012; Omokhafe and Emuedo, 2006). When *E.ulmoides* seedlings were subjected to drought treatment for 10 days, the expression levels of *EuUSP1*, *EuUSP16*, *EuUSP22*, and *EuUSP23* were significantly upregulated. This change coincided with the alteration in *E.ulmoides* rubber content observed after 10 days of drought treatment. Furthermore, research indicates that temperature can influence *E.ulmoides* rubber synthesis (Yao and Zhao, 2023). In this study, during low-temperature treatment for 10 days, the expression levels of *EuUSP1*, *EuUSP6*, *EuUSP7*, *EuUSP16*, *EuUSP18*, and *EuUSP20* were upregulated in the stems of *E.ulmoides* seedlings but downregulated in the leaves. This expression pattern aligns with the observed increase in rubber content in the stems and the decrease in rubber content in the leaves following treatment. Collectively, these findings suggest that *EuUSP16* may be involved in *E.ulmoides* rubber synthesis under stress conditions.

### 4.3 Relationship between *EuUSP16* gene expression and *E.ulmoides* rubber biosynthesis

*E.ulmoides* rubber biosynthesis primarily occurs via two metabolic pathways: the MVA pathway and the MEP pathway. Numerous key enzymes function within these pathways. Geranylgeranyl diphosphate synthase (GGPPS) and farnesyl diphosphate synthase (FPS) synthesize GGPP and FPP, respectively, providing the isoprenoid precursor isopentenyl diphosphate (IPP) for rubber chain elongation (Miziorko, 2011). Rubber elongation factor (REF) binds to the rubber synthase complex and directly participates in isoprenoid chain elongation, while small rubber particle protein (SRPP) stabilizes rubber particle structure, preventing polymer degradation. In *E.ulmoides* adventitious buds overexpressing *EuUSP16*, the relative expression levels of *EuGGPPS*, *EuFPS1*, *EuREF*, and *EuSRPP1* genes were significantly increased. Conversely, in *EuUSP16*-silenced *E.ulmoides* plants, the relative expression levels of *EuFPS1*, *EuREF*, and *EuSRPP1* genes were significantly decreased. Furthermore, rubber content was significantly elevated in *EuUSP16*-overexpressing callus tissues. These findings strongly indicate that *EuUSP16* positively regulates the expression of core rubber biosynthesis genes (*EuFPS1*, *EuREF*, *EuSRPP1*). Whether *EuUSP16* promotes precursor accumulation needs to be verified by detecting IPP levels in *EuUSP16*-transgenic overexpression plants. We also observed that Isopentenyl diphosphate isomerase(IPI)gene *EuIPI* expression was significantly downregulated in *EuUSP16*-overexpressing adventitious buds, while its expression was significantly upregulated in *EuUSP16*-silenced plants. IPI catalyzes the interconversion of IPP and dimethylallyl diphosphate (DMAPP) (Liao et al., 2016). The downregulation of the *EuIPI* gene may reduce the conversion of IPP to DMAPP, thereby diverting more IPP towards rubber synthesis rather than the synthesis of other metabolic products. Whether *EuUSP16* promotes precursor accumulation needs to be verified by detecting IPP levels in *EuUSP16*-transgenic overexpression plants. Simultaneously, the protein-protein interaction between EuUSP16 and rubber synthase requires verification via yeast two-hybrid (Y2H) assay.

### 4.4 The EuDof transcription factor regulates the expression of the *EuUSP16* gene

Studies have shown that the presence of specific *cis*-acting elements within promoter regions can regulate tissue-specific gene expression (Chen et al., 2014, 2015; Rusconi et al., 2013). With the exception of P_EuUSP1-4_, *GUS* expression levels were highest in leaves. For P_EuUSP16-1_, GUS expression showed no significant difference between roots and leaves. This may be associated with the presence of drought-inducible response elements in P_EuUSP16-1_, as drought primarily results from soil water deficit. Plant roots, being the primary organs for water and nutrient uptake from soil, play a crucial role under drought conditions (Kim et al., 2020). The *GUS* activity driven by P_EuUSP16–1_ was significantly higher than that by P_EuUSP16-2_, indicating that the region from -1967 bp to -1286 bp positively regulates *GUS* expression. Alterations in *cis*-elements within promoter regions can modulate the activity of downstream gene expression. The distance of *cis*-acting elements from the transcription start site (TSS) can influence promoter activity; *cis*-elements located further upstream of the TSS may exhibit reduced activity due to steric hindrance limiting transcription factor binding (Imoto et al., 2008; Kiesenhofer et al., 2018; Rushton, 2016). Following low-temperature treatment of transgenic tobacco plants harboring P_EuUSP16–1_ or P_EuUSP16-2_, the relative *GUS* expression increased in both, but the increase was more pronounced in P_EuUSP16-2_. Conversely, ABA treatment of transgenic tobacco plants carrying P_EuUSP16-1_, P_EuUSP16-2_, or P_EuUSP1–3_ resulted in decreased relative *GUS* expression, with a more significant reduction observed in P_EuUSP16–3_ compared to P_EuUSP16–1_ and P_EuUSP16-2_. These differential responses may be related to the positions of low-temperature and ABA-responsive elements within their respective promoter regions.

Research indicates that Dof transcription factors participate in abiotic stress responses in numerous plant species (Waschburger et al., 2023; Yanagisawa, 2004; Zou and Sun, 2023). ZmDof22 enhances drought tolerance in maize by positively regulating genes involved in stomatal closure to reduce water loss and activating antioxidant enzymes such as SOD and POD (Cao et al., 2024). In cotton, overexpression of *GhDof1* improves salt and cold tolerance (Su et al., 2017). *VaDof17d* enhances cold tolerance in grapevine (Wang et al., 2021). Overexpression of tomato *SlCDF1* and *SlCDF3* genes in Arabidopsis confers enhanced drought and salt tolerance (Corrales et al., 2014). Furthermore, Dof transcription factors play significant regulatory roles in plant secondary metabolism (Guo et al., 2019). The Cys2/Cys2 zinc finger domain is a common DNA-binding domain widely involved in transcriptional regulation. Under drought/cold stress, EuDof directly binds the *EuUSP16* promoter to activate expression, whereas ABA suppresses this activation. This EuDof-*EuUSP16* regulatory pathway contrasts with established TF networks, such as the ethylene-induced HbERF1-mediated upregulation of *HbREF/HbSRPP* in H.brasiliensis, as it operates independently of Jasmonic Acid (JA)/ethylene signaling (Shi et al., 2016; Wu et al., 2010). This identification of a EuDof-*EuUSP16* regulatory axis represents a novel mechanism within plant secondary metabolism. However, the identification of the EuDof transcription factor and its interaction with the *EuUSP16* promoter suggest the potential existence of a complex transcriptional regulatory network. Future studies should further explore interactions between EuDof and other transcription factors, as well as its specific functional role in *E.ulmoides* rubber biosynthesis.

## 5 Conclusion

This study identified 29 *EuUSPs* genes at the genomic level. Among them, *EuUSP16* plays a critical role in *E.ulmoides* rubber biosynthesis. The EuDof transcription factor enhanced the promoter activity of *EuUSP16*. Furthermore, binding of EuDof to the *EuUSP16* promoter was enhanced under low temperature and drought conditions, but inhibited by ABA treatment.

## Data Availability

The datasets presented in this study can be found in online repositories. The names of the repository/repositories and accession number(s) can be found in the article/**Supplementary Material**.
