# Correction: Genome-wide identification of *EuUSPs* in *Eucommia ulmoides* and the role of *EuUSP16* in rubber biosynthesis

**DOI:** 10.3389/fpls.2025.1689576

**Published:** 2025-09-03

**Authors:** Frontiers Production Office

**Affiliations:** Frontiers Media SA, Lausanne, Switzerland

**Keywords:** *Eucommia ulmoides* Oliv., EuUSPs gene family, environmental stress, rubber biosynthesis, EuDof

There was a mistake in **Figure 6** as published. The data for the expression levels of the *EuUSP1* and *EuUSP6* genes in the stem were incorrectly pasted, leading to an error in the figure image. The corrected **Figure 6** appears below.

**Figure 6:**
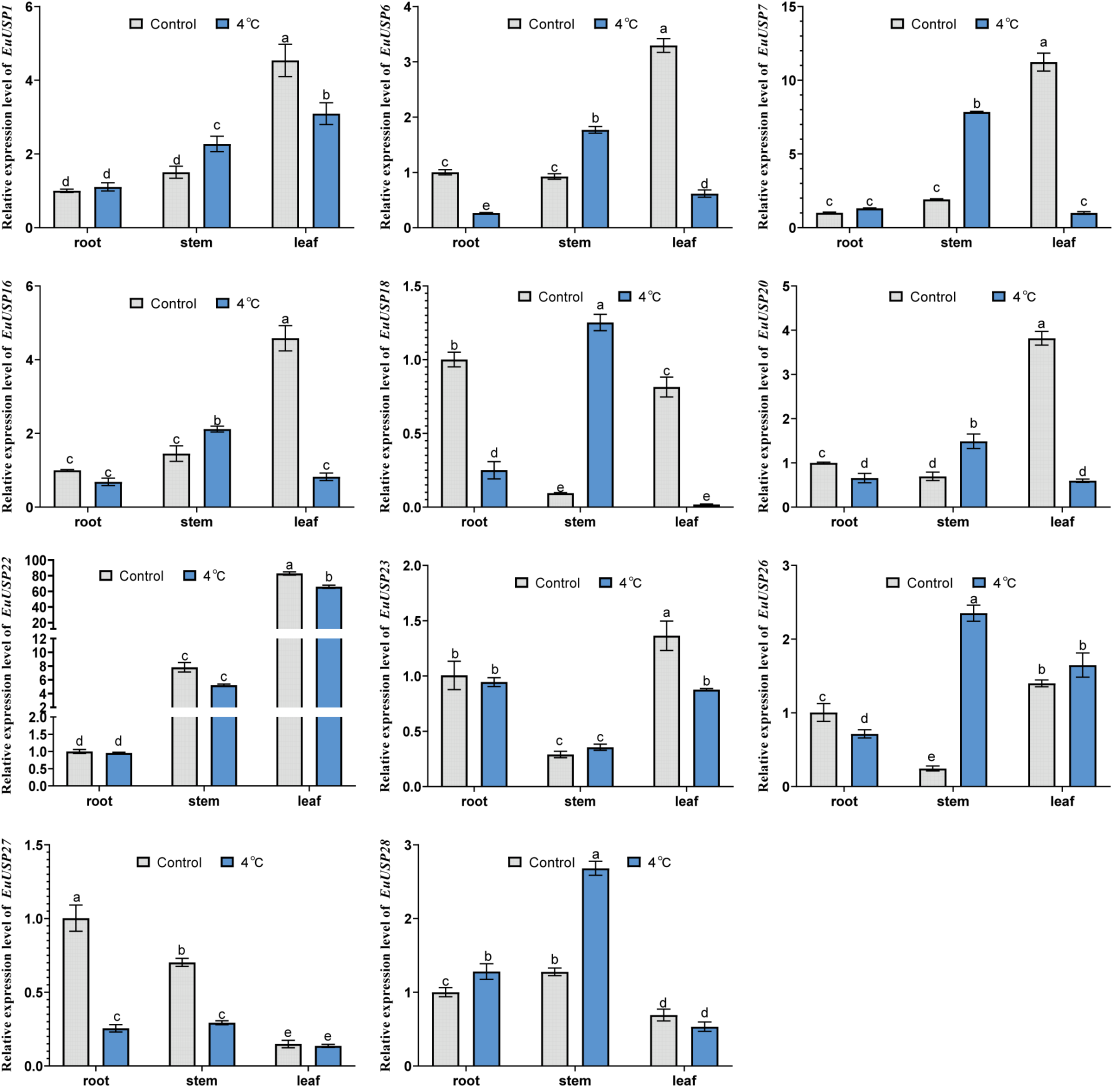
The expression analysis of *EuUSPs* gene in after 4°C treatment. Control: 25°C treatment. Different lowercase letters indicate significant differences (*P*<0.05).

The original version of this article has been updated.

