# Supplementary figures and images for: Genome-wide identification of EuUSPs in Eucommia ulmoides and the role of EuUSP16 in rubber biosynthesis
Source: Front Plant Sci. 2025 Aug 20;16:1655155. doi: 10.3389/fpls.2025.1655155 (PMC12404981; doi:10.3389/fpls.2025.1655155)

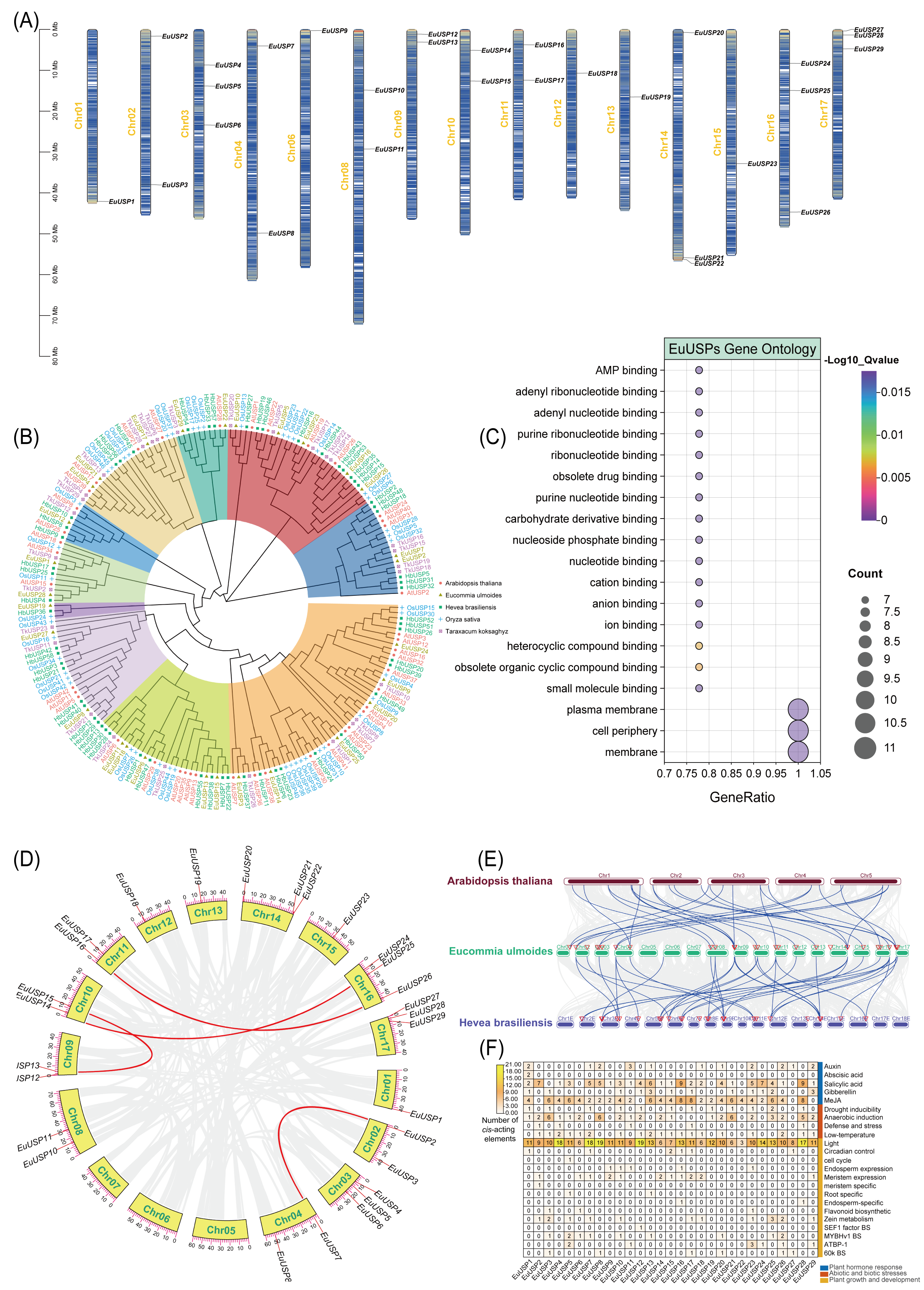

Supplement: Supplementary file 1 [file DataSheet1.zip › Figure/Figure 1.tif]

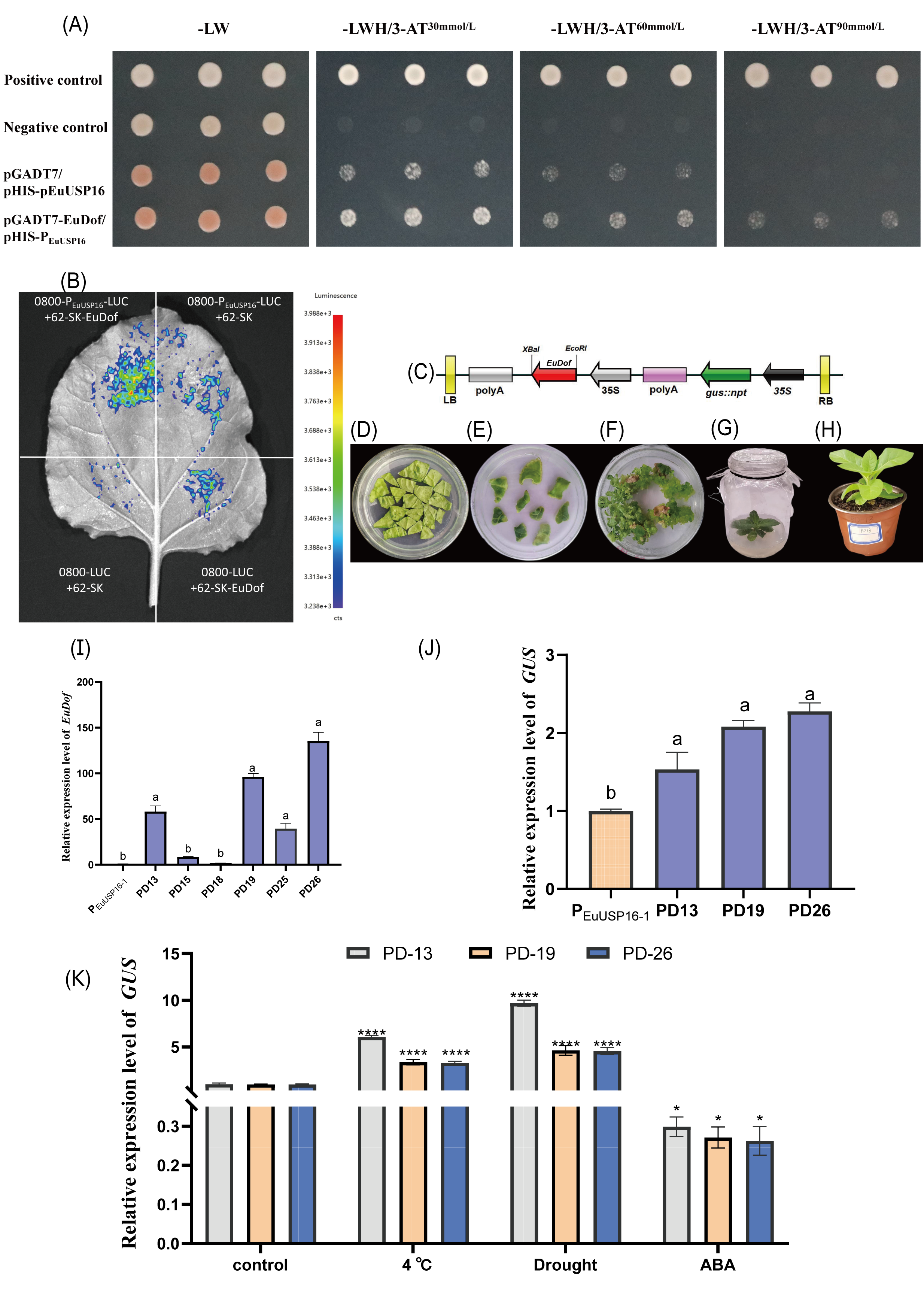

Supplement: Supplementary file 1 [file DataSheet1.zip › Figure/Figure 10.tif]

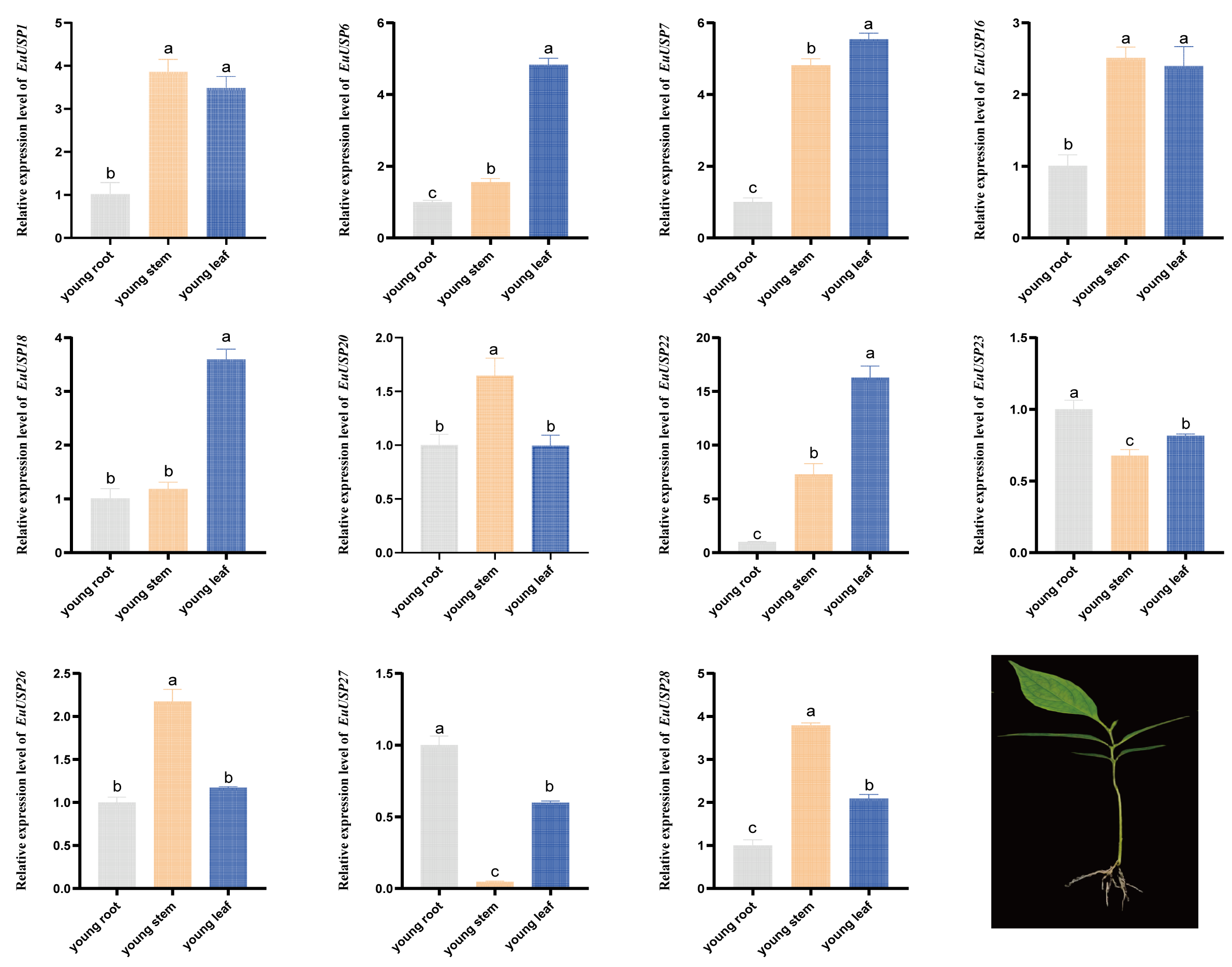

Supplement: Supplementary file 1 [file DataSheet1.zip › Figure/Figure 2.tif]

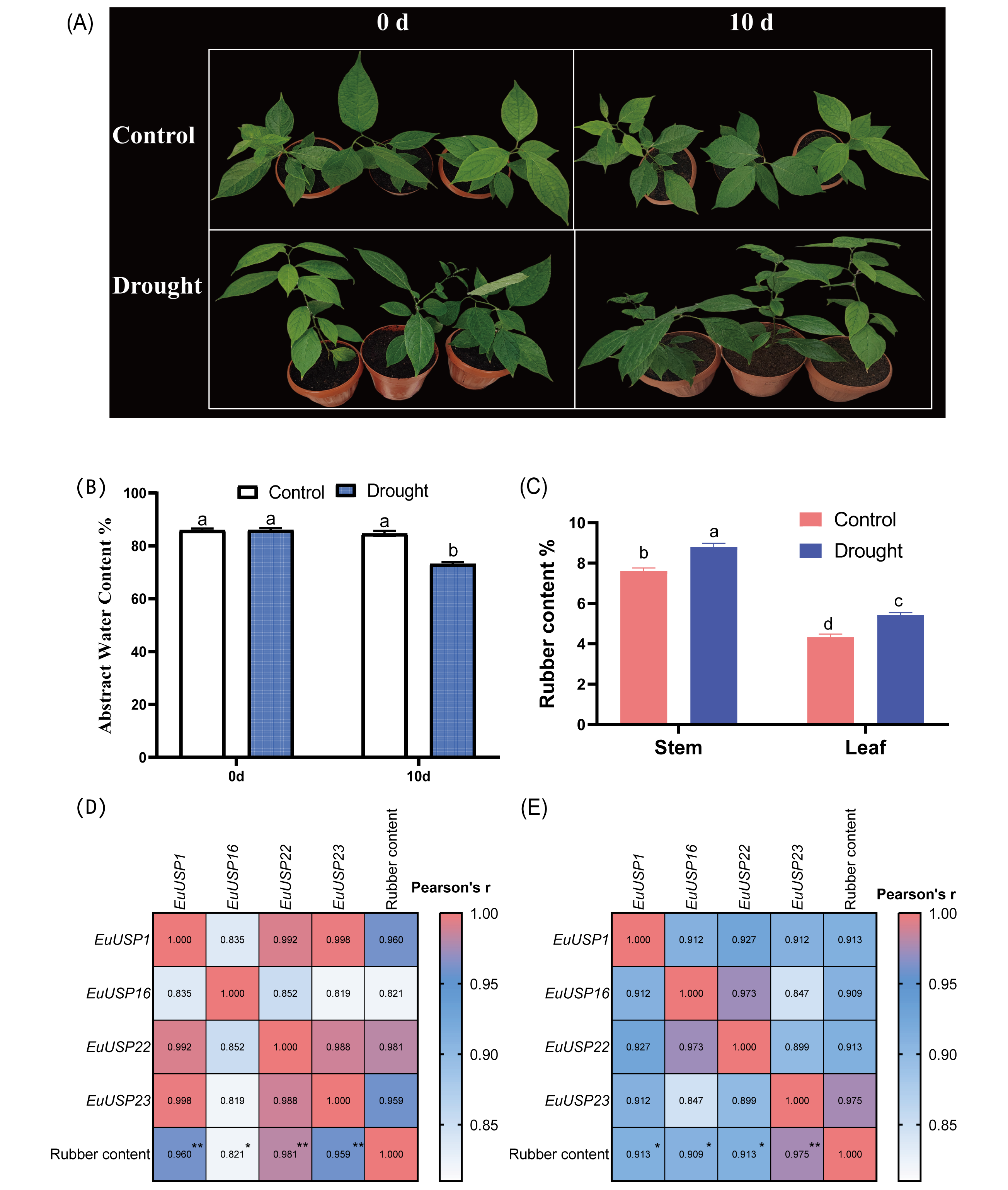

Supplement: Supplementary file 1 [file DataSheet1.zip › Figure/Figure 3.tif]

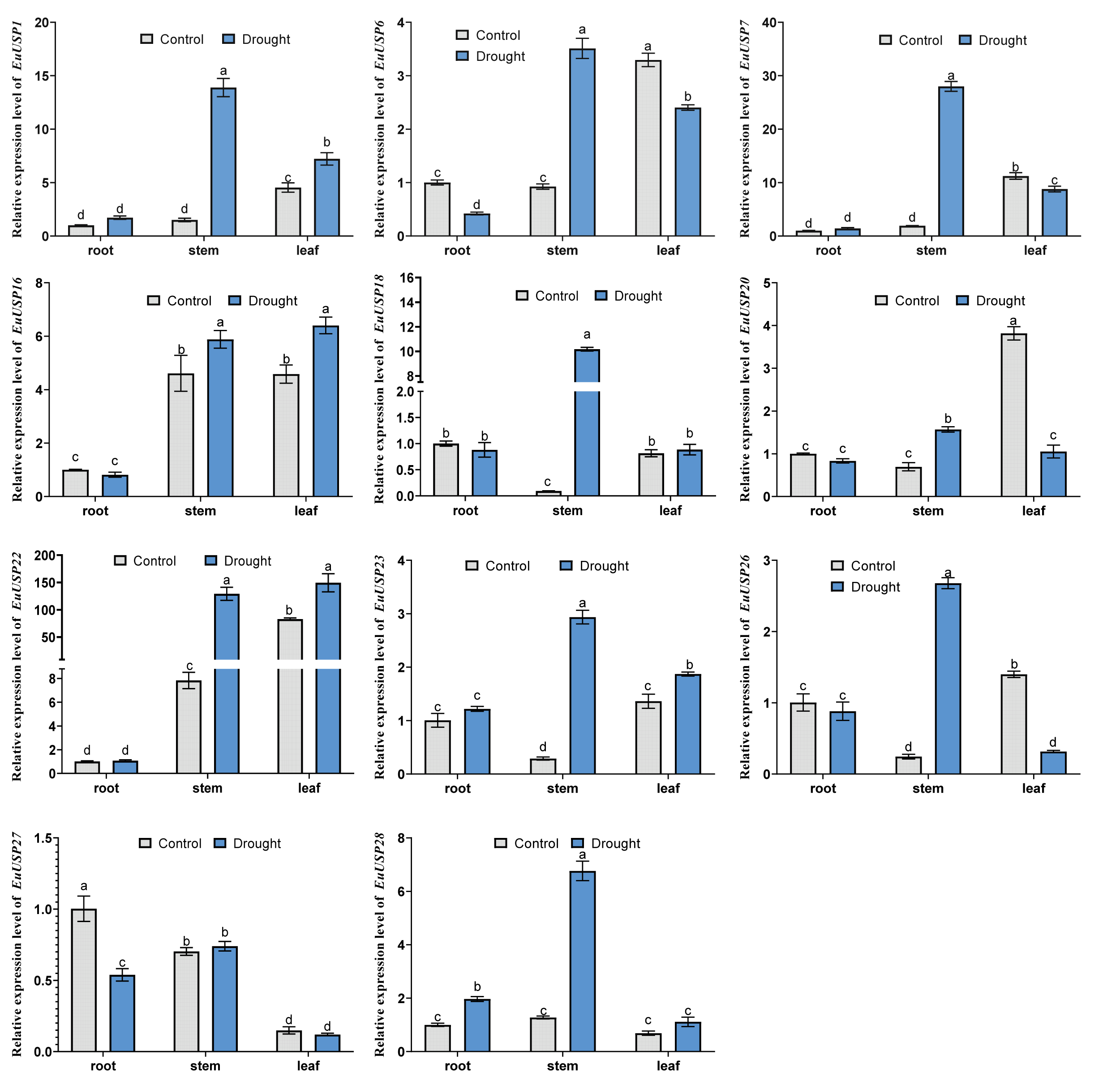

Supplement: Supplementary file 1 [file DataSheet1.zip › Figure/Figure 4.tif]

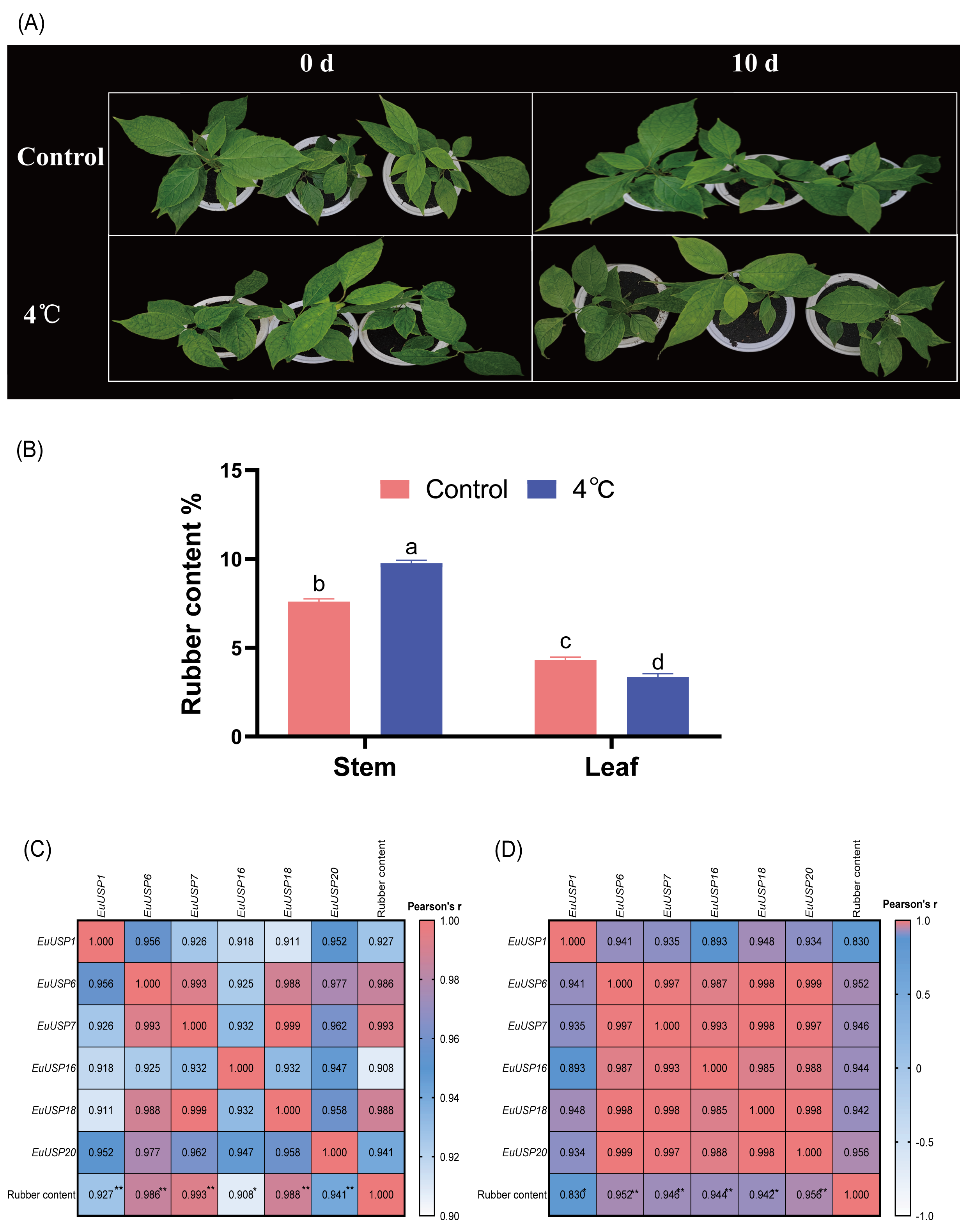

Supplement: Supplementary file 1 [file DataSheet1.zip › Figure/Figure 5.tif]

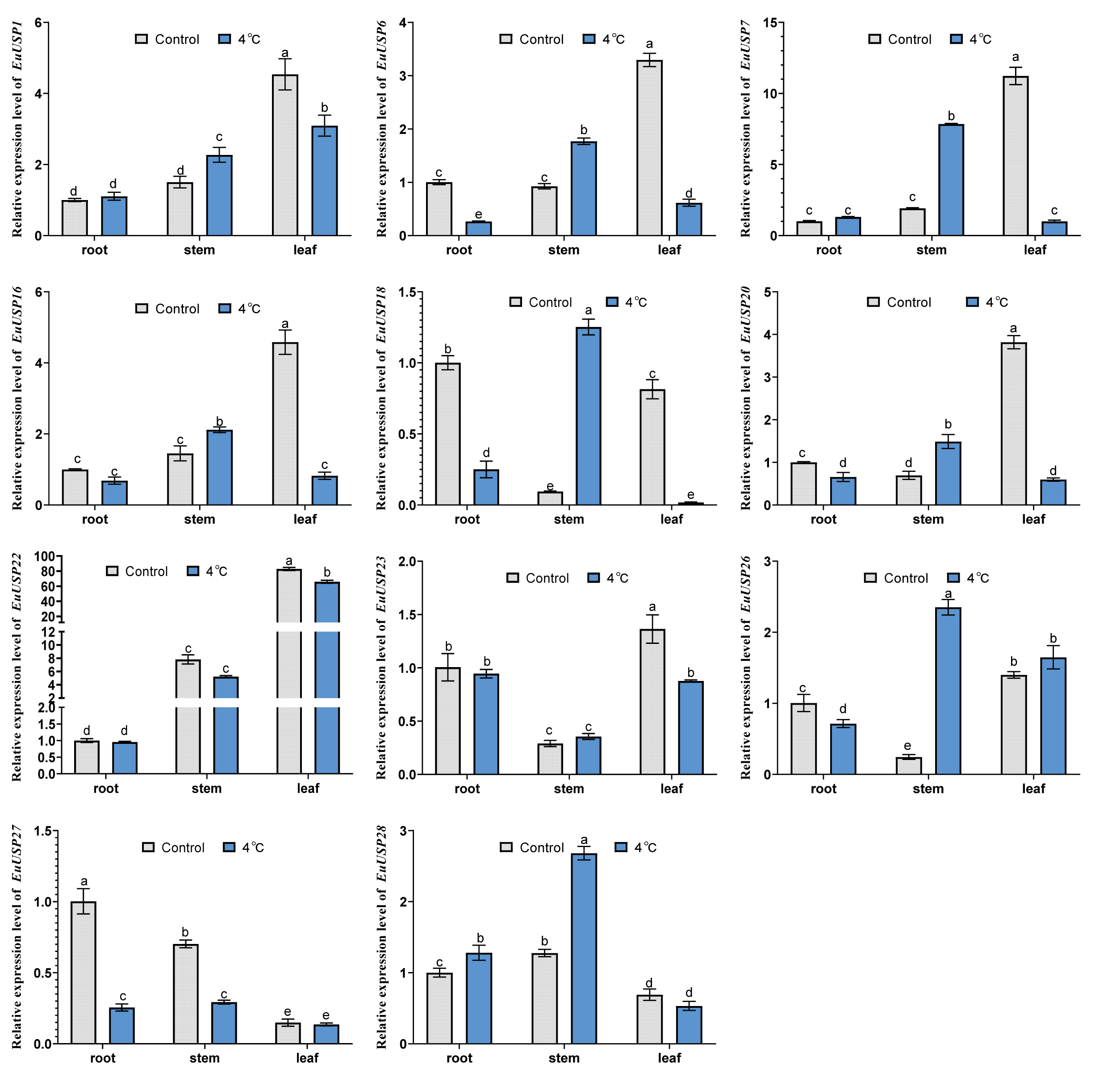

Supplement: Supplementary file 1 [file DataSheet1.zip › Figure/Figure 6.tif]

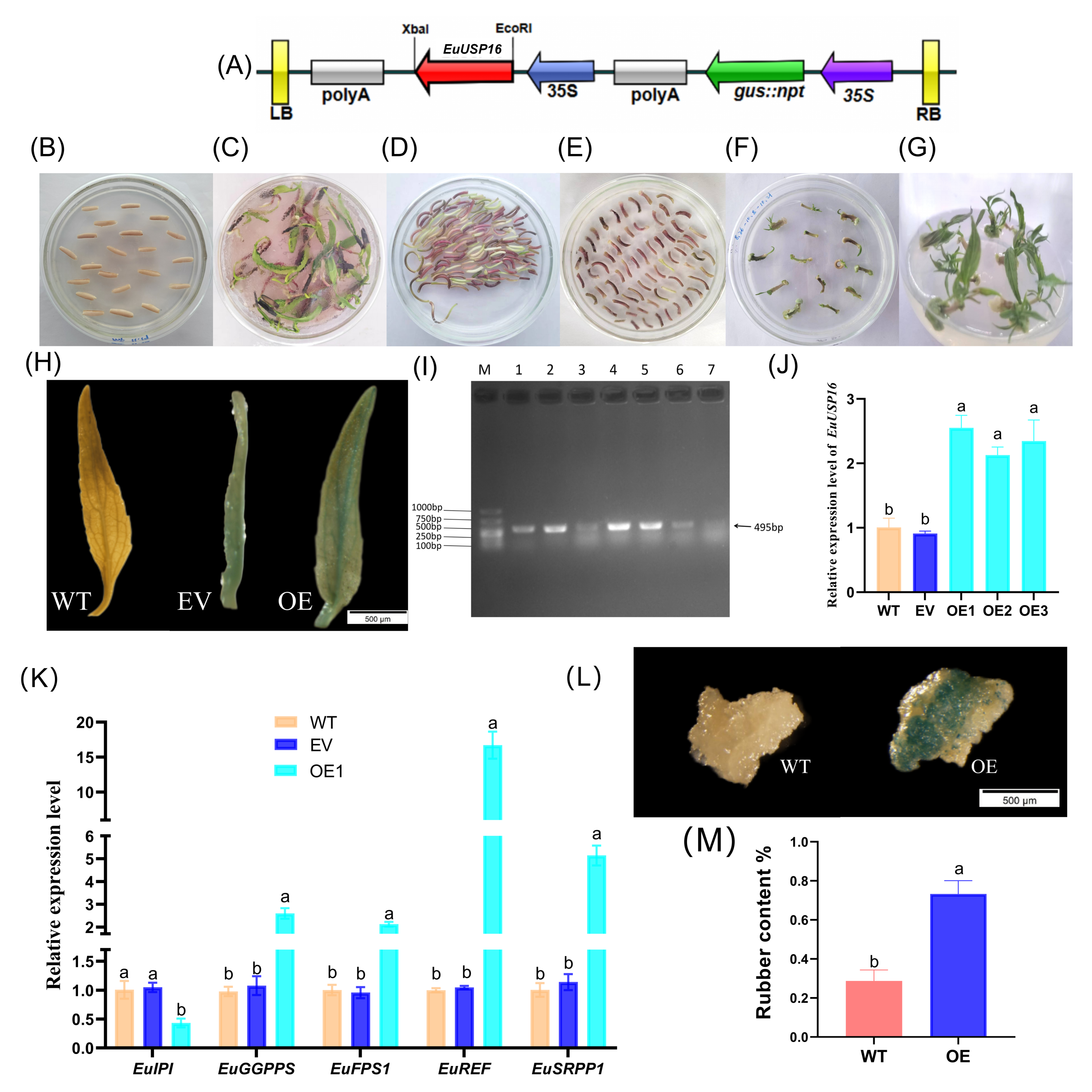

Supplement: Supplementary file 1 [file DataSheet1.zip › Figure/Figure 7.tiff]

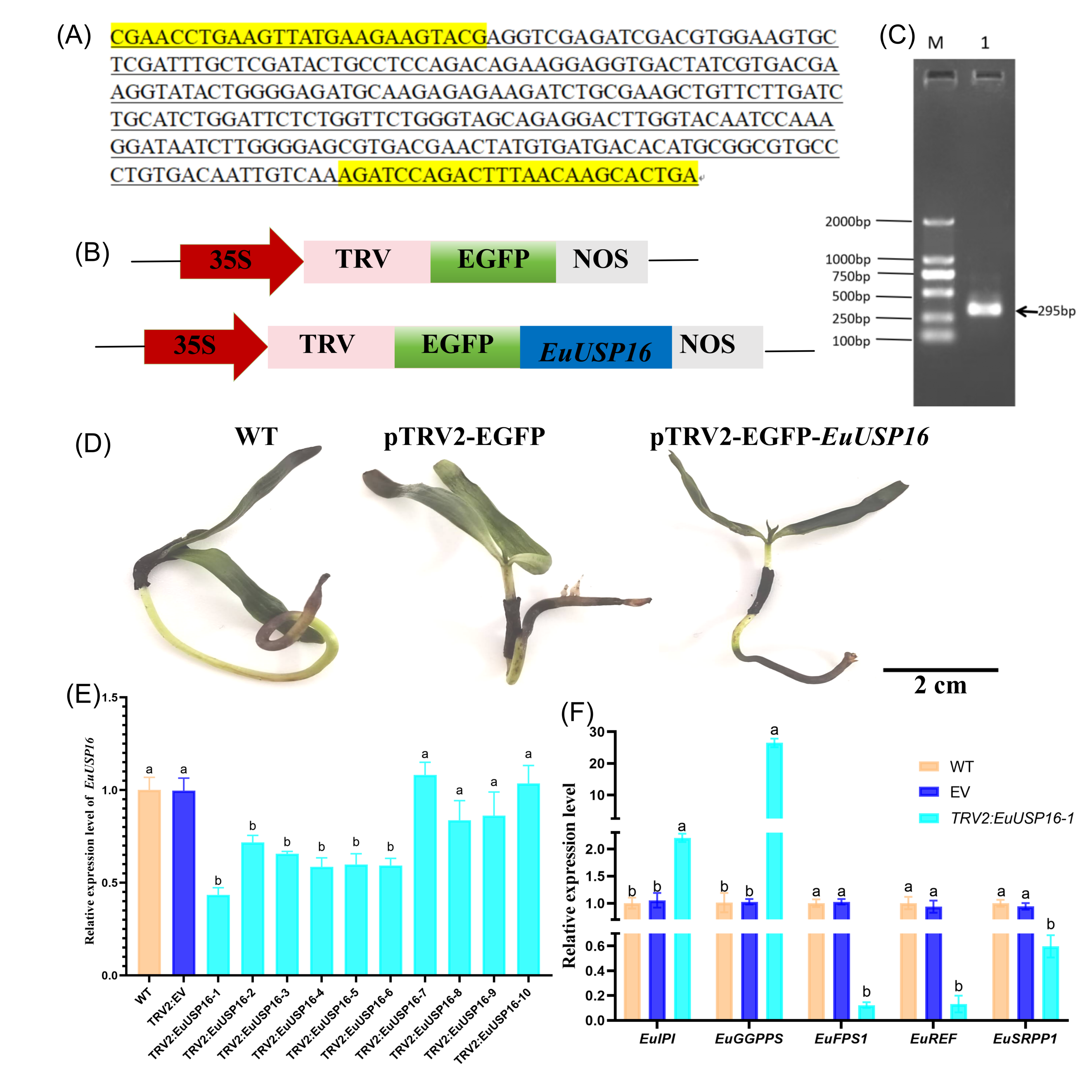

Supplement: Supplementary file 1 [file DataSheet1.zip › Figure/Figure 8.tiff]

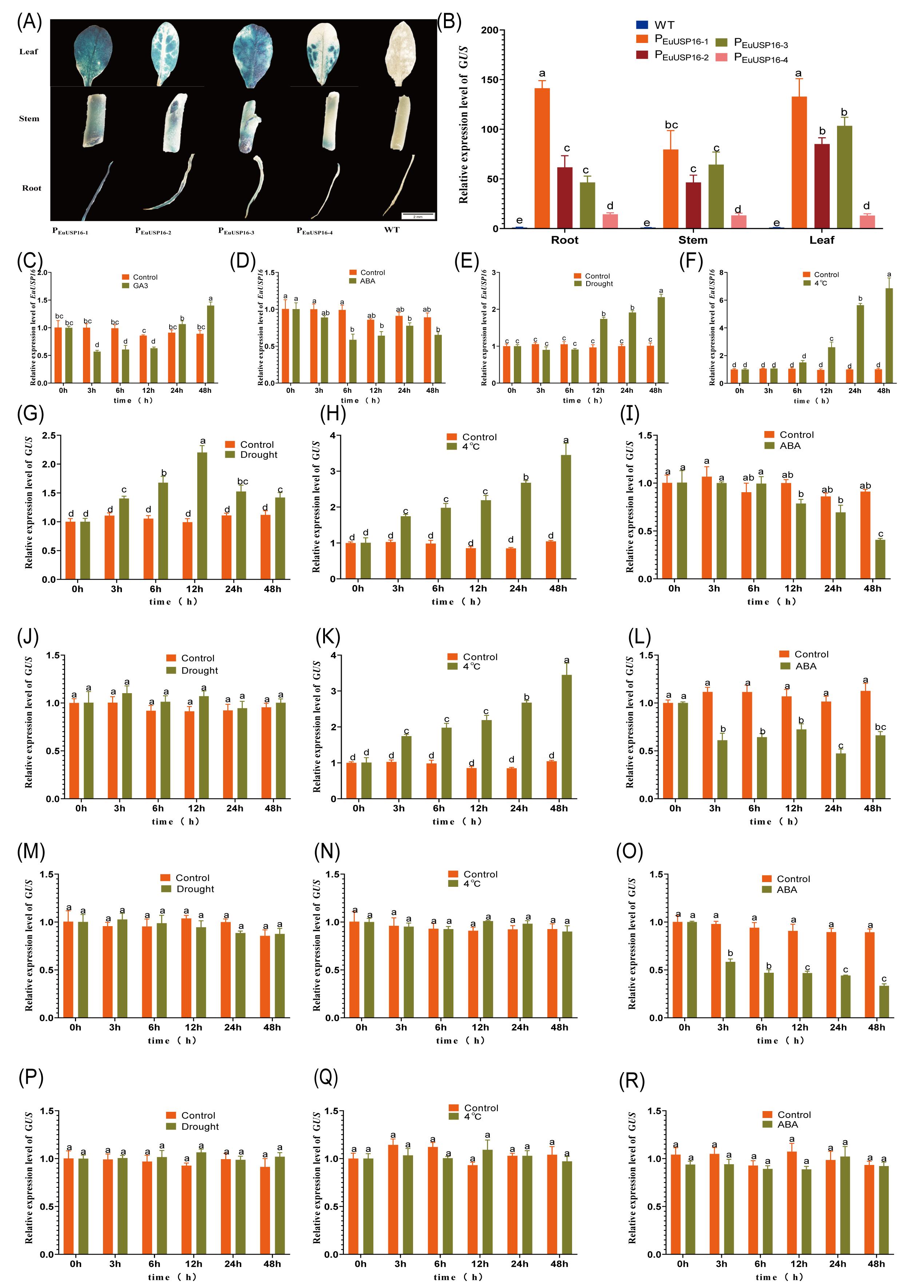

Supplement: Supplementary file 1 [file DataSheet1.zip › Figure/Figure 9.tif]

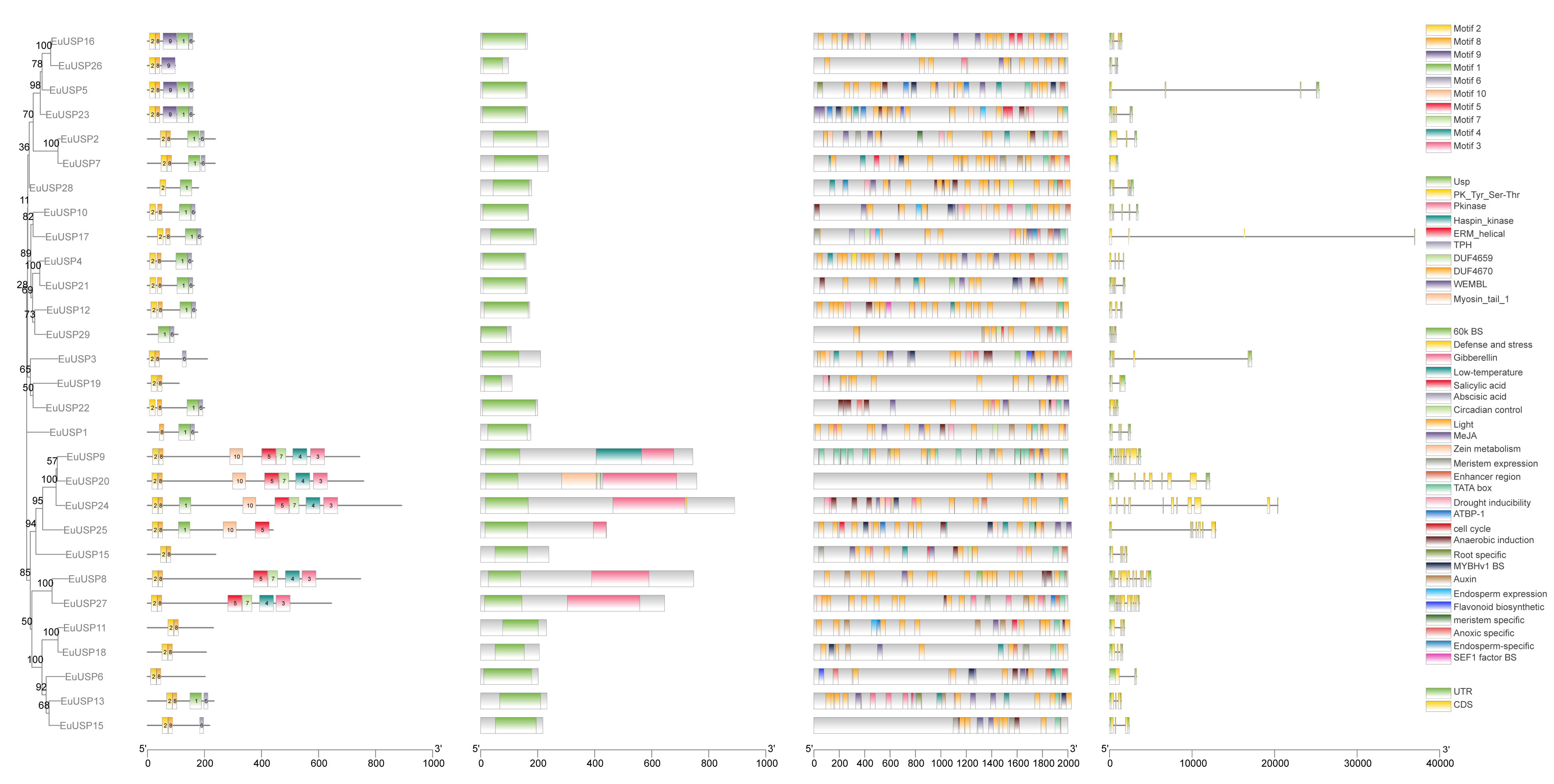

Supplement: Supplementary file 1 [file DataSheet1.zip › Figure/Supplementary Figure 1.tiff]
